# Nested Insertions and Accumulation of Indels Are Negatively Correlated with Abundance of *Mutator*-Like Transposable Elements in Maize and Rice

**DOI:** 10.1371/journal.pone.0087069

**Published:** 2014-01-27

**Authors:** Dongyan Zhao, Ning Jiang

**Affiliations:** Department of Horticulture, Michigan State University, East Lansing, Michigan, United States of America; Georgia Institute of Technology, United States of America

## Abstract

*Mutator*-like transposable elements (MULEs) are widespread in plants and were first discovered in maize where there are a total of 12,900 MULEs. In comparison, rice, with a much smaller genome, harbors over 30,000 MULEs. Since maize and rice are close relatives, the differential amplification of MULEs raised an inquiry into the underlying mechanism. We hypothesize this is partly attributed to the differential copy number of autonomous MULEs with the potential to generate the transposase that is required for transposition. To this end, we mined the two genomes and detected 530 and 476 MULEs containing transposase sequences (candidate coding-MULEs) in maize and rice, respectively. Over 1/3 of the candidate coding-MULEs harbor nested insertions and the ratios are similar in the two genomes. Among the maize elements with nested insertions, 24% have insertions in coding regions and over half of them harbor two or more insertions. In contrast, only 12% of the rice elements have insertions in coding regions and 19% have multiple insertions, suggesting that nested insertions in maize are more disruptive. This is because most nested insertions in maize are from LTR retrotransposons, which are large in size and are prevalent in the maize genome. Our results suggest that the amplification of retrotransposons may limit the amplification of DNA transposons but not vice versa. In addition, more indels are detected among maize elements than rice elements whereas defects caused by point mutations are comparable between the two species. Taken together, more disruptive nested insertions combined with higher frequency of indels resulted in few (6%) coding-MULEs that may encode functional transposases in maize. In contrast, 35% of the coding-MULEs in rice retain putative intact transposase. This is in addition to the higher expression frequency of rice coding-MULEs, which may explain the higher occurrence of MULEs in rice than that in maize.

## Introduction

Transposable elements (TEs) are genomic sequences that are capable of moving from one position to another. Based on the transposition intermediate, TEs can be divided into two classes. Class I, also called RNA or retrotransposons, transpose via an RNA intermediate. Class II, DNA transposons, transpose via a DNA intermediate. TEs can also be grouped into autonomous or non-autonomous elements, where the former encode proteins (transposase for class II elements) responsible for the transposition of themselves as well as their corresponding non-autonomous counterparts.

TEs constitute large fractions of most plant genomes sequenced to date, *i.e.*, ∼85% of maize [1], ∼62% of soybean [2] and sorghum [3], ∼63% of tomato [4], ∼43% of papaya [5], and ∼35% of rice genomes [6]. Comparative analysis reveals that the abundance of different classes of TEs (*e.g.*, RNA and DNA TEs) varies dramatically in different plants. In rice, the genomic coverage of RNA TEs (20%) is 1.5-fold of that of DNA TEs (13%) whereas in maize the difference is 8-fold between RNA (76%) and DNA (9%) TEs [1], [6]. This difference is the greatest in the papaya genome, which contains ∼43% RNA TEs and very few DNA TEs (0.2%). In general, the amount of retrotransposons is correlated with plant genome size whereas such correlation is not found for DNA elements [7].

*Mutator*-like transposable elements (MULEs), first discovered in maize [8], [9], are widespread in plants [10], [11], fungi [12], [13], and animals [14]. MULEs are one of the most complex TE families, with dramatic variation in structure, sequence, size, and abundance within a genome and among different plant genomes [1], [10], [15]. Based on the similarity of their terminal inverted repeats (TIRs), MULEs can be grouped into TIR-MULEs or non-TIR-MULEs, where TIR-MULEs are characterized by long TIRs (100–500 bp) with high sequence similarity and non-TIR-MULEs have relatively short TIRs with low sequence similarity between their terminal sequences [10]. Within the TIR-MULE group, a special subgroup was discovered in several plants, which contains tandem TIRs (two consecutive TIRs) flanking the internal sequence [15]. In addition, some MULEs harbor gene and/or gene fragments, referred to as Pack-MULEs [16]. Their high transposition frequency combined with capability to duplicate gene fragments suggest that MULEs play important roles in genome evolution [17], [18]. Individual genomes can contain all forms of MULEs, including TIR- and tandem TIR-MULEs, non-TIR-MULEs, and Pack-MULEs, albeit with differential abundance [10], [15]. Rice by far contains the largest amount of known MULEs (n = 32,000, 5.5% of the genome) and Pack-MULEs (n = 2,924) [15], [19]. As a close relative, maize contains 12,900 MULEs (1% of the genome) and 276 Pack-MULEs, which are relatively less abundant given that its genome size is over 5 times larger than rice (2,066 Mb vs. 370 Mb) [1], [6]. The papaya genome represents an extreme case, which is almost void of DNA TEs including MULEs. Despite the prevalence and importance of MULEs, the mechanism underlying the differential amplification of these elements or DNA elements in general remains largely unknown.

The first autonomous *Mutator* element discovered is *MuDR* in maize. It encodes two proteins, *MURA*, the major protein responsible for its transposition, and *MURB*, a helper protein found only in the *Zea* genus and for which the function is still unclear [17], [20], [21]. MULE transposases belong to the DDE transposase family, which commonly consists of a helix-turn-helix (HTH) DNA-binding domain at the amino terminus and a DDE catalytic domain at the carboxyl terminus [22], [23]. *MURA*-like transposase sequences have been discovered in many other organisms, including plants, fungi, and animals [24]–[26]. In addition to the differences in TIR length, *MURA*-like transposase sequences are also divergent among MULEs as demonstrated by distinct subfamilies [11], [25]. Furthermore, some MULE transposase sequences, such as *FAR1* and *MUSTANG*, have been domesticated as cellular genes and are no longer associated with any mobility [27], [28].

The abundance of TEs is a result of the interplay between the amplification through transposition, duplication, horizontal transfer and loss via excision, sequence erosion, deletion *etc.* [29]. Since autonomous elements are responsible for the transposition of both themselves and their corresponding non-autonomous counterparts, their abundance and activity influence the speed of amplification, and therefore contribute to the abundance of TEs in a genome. Maize and rice belong to the same family (*Poaceae*) with a common ancestor occurring 50–70 million years ago [30]. As the most important crops worldwide, the genome of both maize and rice were sequenced through a hierarchical method using bacterial artificial chromosome clones (BACs) accompanied by high-density genetic maps [1], [6]. The availability of high quality genomic sequence and well-annotated TEs allow a comparative study of TEs in these two organisms. In this study, we report the detection and analysis of all MULEs containing transposase sequences (candidate coding-MULEs) in the maize and rice genomes. In addition, we dissected the possible factors involved in the loss of coding capacity of those elements, which facilitate the understanding about the underlying mechanisms for differential abundance of MULEs in the two genomes.

## Materials and Methods

### Genomic sequences and TE libraries

The B73 maize genomic sequence RefGen_v2 was downloaded from the MaizesSquence.org (http://www.maizesequence.org/) and the Nipponbare rice genomic sequence Release 7 was downloaded from the Rice Genome Annotation Project at Michigan State University (http://rice.plantbiology.msu.edu/index.shtml). TE library for rice and MULE TIR libraries (MULE_TIR) for both maize and rice were constructed and curated by the Jiang Lab. The TE library for maize was downloaded from the Maize Transposable Element Database (http://maizetedb.org/~maize/) in August 2011.

### Identification of candidate coding-MULEs

To maximize the possibility of detecting coding-MULEs, all the transpositionally active *MURA*-related transposases (*e.g. MURA*, *Hop*, *Jittery*, *Os3378*, *AtMu1*) [8], [9], 12,24,26,31 and some *MURA*-related transposases with conceptual translations were collected and used in the NCBI TBLASTN search (e <10^−5^, http//www.ncbi.nlm.nih.vov/blast/) against the maize and rice genomes, respectively. Detailed information about the MULEs used as queries is provided in Table S1. Sequences producing significant alignments (e <10^−5^) were retained and their 50 kb flanking sequences were retrieved. The resulting sequences were masked by the MULE_TIR libraries of maize and rice, respectively, using the RepeatMasker program (www.repeatmasker.org). A candidate coding-MULE must satisfy the following criteria. First, the pairwise identity between its two TIRs should be higher than 75% and in an inverted orientation with the TIR-ends facing outwards. Second, sequence homologous to transposases (NCBI BLASTX, e <10^−5^) must be located within the two TIRs. Lastly, a TSD (8–11 bp) immediately flanking the TIRs must be present. For TSDs of 8 bp, one mismatch or indel (one nucleotide) is allowed and for those equal or larger than 9 bp, a maximum of 2 mismatches (or one mismatch plus one indel) is allowed.

### Detection of nested TE insertions in the candidate coding-MULEs and estimation of ages of nested LTR retroelements

The candidate coding-MULEs were masked using the maize and rice TE libraries, respectively, using the RepeatMasker program (www.repeatmasker.org) and the resulting output file was used to determine the number and type of nested TEs in the candidate coding-MULEs. The coordinates of the coding regions (as defined in the following section) and the inserted TEs were compared in order to determine whether the inserted TEs interrupt the open reading frame of the candidate coding-MULEs. If the coordinates of the inserted TE are within the coordinates of the coding region, the TE is considered to be inserted in the coding region of the candidate coding-MULE.

Estimation of the insertion time of intact long terminal repeat (LTR) retrotransposons was based on the divergence of the two LTRs. Sequences of the two LTRs of one element were aligned using the MUSCLE program [32] and the number of substitutions per site between the two LTRs was obtained using MEGA 5.10. Nucleotide substitution rate 1.3×10^−8^ per site per year generated by Ma *et al.* [33] was used to calculate the approximate age of the LTR elements.

### Determination of the coding capacity of the candidate coding-MULEs

To determine the coding region of the candidate coding-MULEs, nested insertions were first masked using the maize and rice TE libraries (without MULE sequences), respectively, using the RepeatMasker program (www.repeatmasker.org). The masked sequences were used to search (NCBI BLASTX with e <10^−5^) against the total protein datasets of maize and rice, which contain annotated TE proteins and were downloaded from the MaizeSequence.org (http://www.maizesequence.org/) and the Rice Genome Annotation Project (http://rice.plantbiology.msu.edu/index.shtml), respectively. To ensure that the annotated proteins producing significant alignments (smallest e-value) with the candidate coding-MULEs were indeed MULE transposase, these protein sequences were used to search against the above mentioned *MURA* and *MURA*-related transposase sequences using the NCBI BLASTP (e <10^−5^) program. If the annotated proteins had significant alignments (e <10^−5^) with *MURA* or *MURA*-related sequences, the coding region was defined according to the annotated protein. For the annotated proteins without any significant alignment with known transposases, it suggested that the transposase sequence was not annotated in an open reading frame (ORF) because of some defects (*e.g*., frameshift, premature stop codon, or large deletions). In this case, the nucleotide sequences of the elements were used to search (NCBI BLASTX with e <10^−5^) against the *MURA* and *MURA*-related transposase sequences directly and the regions with significant alignments with those transposase sequences were defined as the coding region. For each candidate coding-MULE, the alignment profiles were manually examined and custom python scripts were used to retrieve the sequences encoding transposases of the candidate coding-MULEs. After obtaining the transposase coding sequences of the candidate coding-MULEs, their coding capacity was evaluated for the presence of DNA binding and catalytic domains. The HTH DNA binding domain was determined using the Jpred3 program (http://www.compbio.dundee.ac.uk/www-jpred/), which was capable of detecting all the experimentally defined HTH domains of some well-annotated transposases (*e.g*., *Hermes*, *Tc3*, *Mos1*, *Phage Mu*) [34]. To locate the DDE domain, a multiple sequence alignment was conducted using the transposase coding sequences of the candidate coding-MULEs in maize and rice separately, with the *MURA* protein sequence as a reference. The conserved catalytic domain was established by comparing candidate coding-MULEs with that of *MURA* and positions of the DDE amino acids in the transposases were recorded. Specifically, if the three amino acids (DDE) are all present, and the length from the first “D” to the “E” is longer than 100 amino acids, without premature stop codon(s) and frameshift(s), the element was considered to contain a catalytic domain.

### Determination of indels in the candidate coding-MULEs

The number and length of indels between two homologous coding-MULEs were determined using the following procedure. First, sequences of the candidate coding-MULEs (after removing nested TEs) were used to conduct an all vs. all search using the NCBI BLASTN program (e <10^−10^). Second, after the self-match was excluded, the element that produced the most significant alignment (the two sequences with the longest alignment) was retained to form a pair with the query coding-MULE, for which pairwise alignment was conducted using the “gap” program available from the GCG package (version 11.0, Accelrys Inc., San Diego, CA). Lastly, the number and length of indels were normalized based on the total length of the two elements to make them comparable between maize and rice. That is, the number of indels was divided by the total length of the two elements, giving a value of the average number of indels per kb (NIK). Similarly, the normalized length of indels is the average length of indels per kb (LIK). Grouping of different coding-MULE pairs was based on two parameters, *i.e.*, pairwise nucleotide identities and synonymous substitution rates (Ks), respectively. To calculate the Ks values, coding sequences (as defined in the previous section) of these element pairs were aligned based on amino acid codons, premature stop codons and frameshifts were removed to achieve correct coding frame. The resulting aligned sequences in fasta format were changed to.axt format using a custom Python script and Ks values were determined using KaKs_Calculator program [35]. Comparison of NIK and LIK of coding-MULE pairs with similar identity or Ks between maize and rice was conducted using the SAS9.3 program at the High Performance Computing Centre, Michigan State University. Nested insertions and one copy of the TSD were excluded upon alignment. The pairs of candidate coding-MULEs used in the alignments were provided in Table S3 and their original sequences were provided in fasta format in Data S1.

In addition, the indel numbers in the LTR sequences of the *Copia*-like LTR retrotransposon in the two genomes were calculated. The sequences of LTRs in rice and maize were from the rice and maize TE libraries mentioned above. The LTR sequences were used to search against the maize and rice genomes using the RepeatMasker program (www.repeatmasker.org). Custom Python scripts were used to extract the coordinates of intact LTRs and retrieve their sequences. Pairwise sequence identity and determination of average indel numbers were similar to that conducted for coding-MULEs.

### Phylogenetic analysis

**Figure 1:**
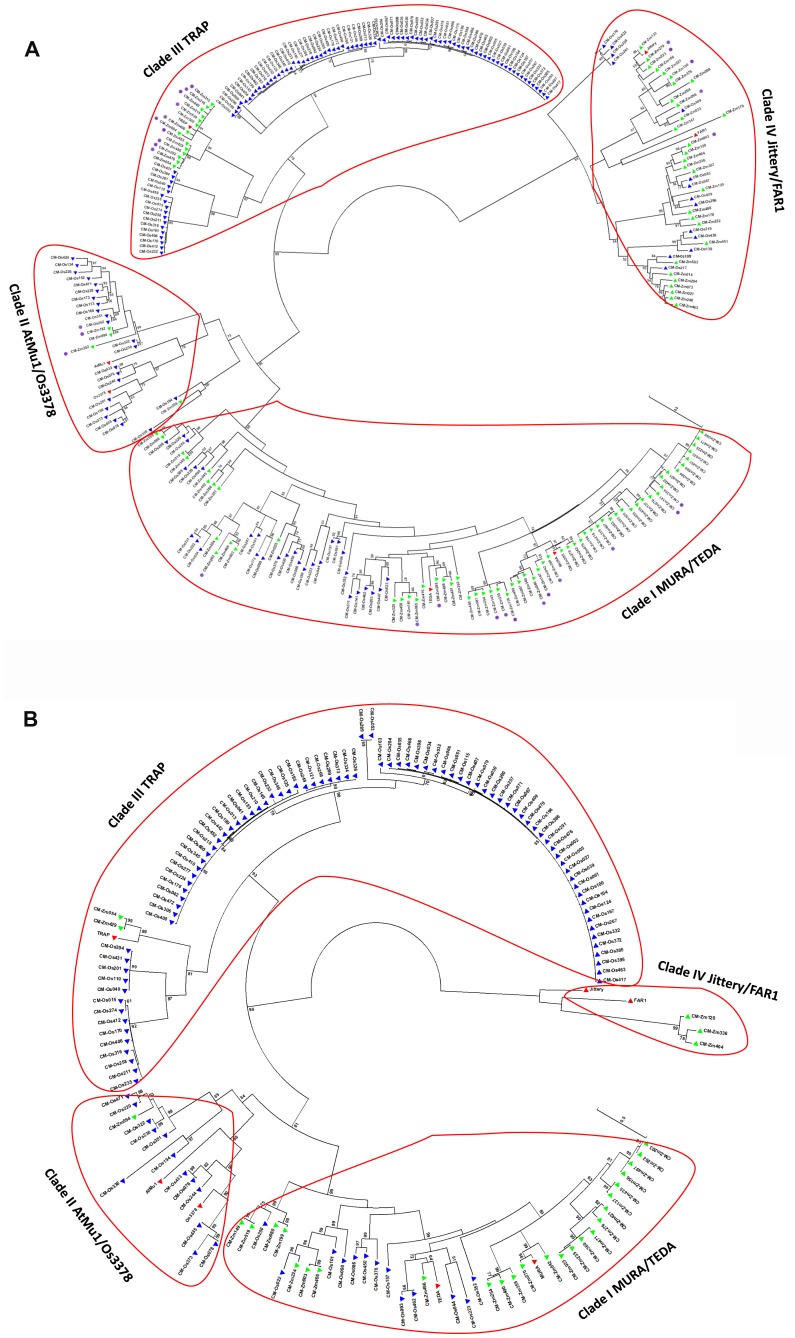
Phylogenetic analysis of the candidate coding-MULEs with DDE domain. A). All representative candidate coding-MULEs containing DDE domain; B). Candidate coding-MULEs with putative intact transposases. Candidate coding-MULEs in maize are denoted by green triangles; those in rice are denoted by blue triangles; known MULE transposases are denoted by red triangles, maize coding-MULEs with nested LTR insertions are denoted with purple round dots.

Amino acid sequences of the catalytic region (corresponding to 331–489 amino acids of *MURA*) of the candidate coding-MULEs were used to conduct the phylogenetic analysis in MEGA 5.2.2 [36]. Frameshifts were corrected and premature stop-codons were excluded to ensure appropriate alignment. Figure 1A contains known MULEs and representative elements with catalytic domains in the two genomes. The representatives were chosen as following: if two elements shared 80% identity in 80% of the element region, only one element was retained. In this way, 211 rice MULEs and 195 maize MULEs with catalytic regions were excluded. Figure 1B contains known MULEs in Figure 1A and all MULEs with putative intact transposase, with the exception of one element family in rice. This element family contains 108 members and 59 of them contain putative intact transposases, and only four representative elements were chosen to be included in Figure 1B (clade III) in order to attain a manageable size. The Maximum Likelihood method was used to infer the evolutionary history and the tree with the highest log likelihood value is shown. A discrete Gamma distribution was used to model evolutionary rate differences among sites (5 categories (+G, parameter  =  4.0540)). All positions with higher than 95% site coverage were used in the analysis and only bootstrap (500 replicates) values equal or higher than 50% were shown.

### Determination of the expression status of the candidate coding-MULEs

Expression Sequence Tags (ESTs) for maize (516,425 ESTs) and rice (1,253,557 ESTs) were downloaded from the NCBI dbEST database (http://www.ncbi.nlm.nih.gov/dbEST/index.html) on January 4^th^, 2013. The full-length cDNA (flcDNA) library for maize (27,455) was downloaded from The Maize Full Length cDNA Project (http://www.maizecdna.org/) on February 2^nd^, 2013 and that for rice (37,139) was from the Knowledge-based Oryza Molecular Biological Encyclopedia (http://cdna01.dna.affrc.go.jp/cDNA/) on Oct 1^st^, 2008. The EST and flcDNA libraries were concatenated into one file (EST-flcDNA) for both maize and rice, respectively. The candidate coding-MULE sequences were searched against the EST-flcDNA library using the NCBI BLASTN program (e <10^−5^). A coding-MULE is considered to have expression evidence if the identity of matched sequence between the coding-MULE and EST/flcDNA is higher than 99.5% over the entire length of the EST/flcDNA.

## Results

### Candidate MULEs containing transposase sequences (coding-MULEs) in maize and rice

**Figure 2:**
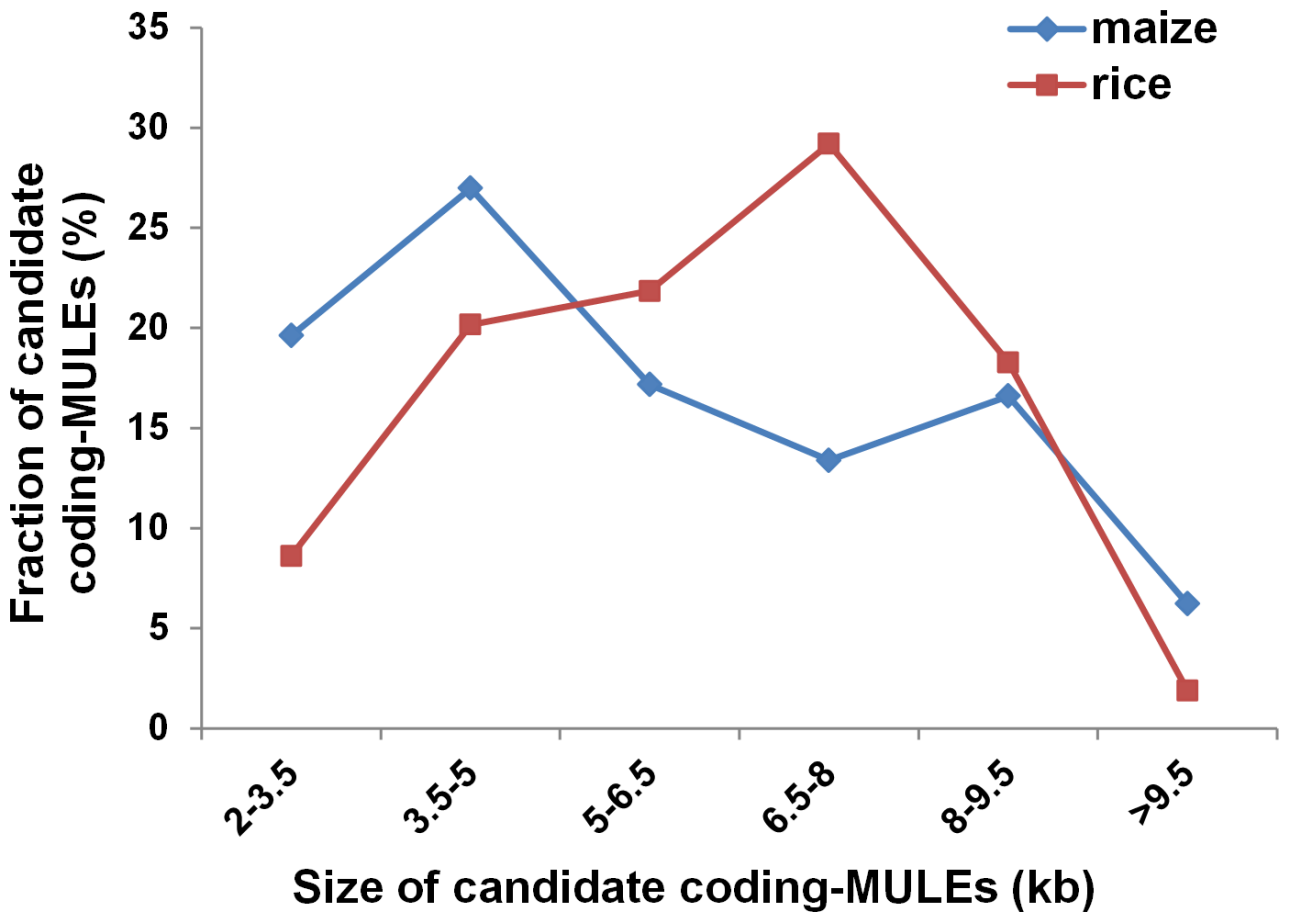
Fraction of candidate coding-MULEs within different size ranges after removing nested TE insertions.

In this study, we focused on TIR-MULEs (see Introduction) containing *MURA*-related transposase sequences (candidate coding-MULEs) in the maize and rice genomes. Non-TIR MULEs (see Introduction) were not considered because of the difficulty in defining the boundary of their termini and target site duplication with high confidence. To retrieve candidate coding-MULEs, a collection of *MURA*-related transposase sequences from several organisms, especially plants (Table S1) was used to search against (NCBI TBLASTN, e<10^−5^) the maize and rice genomic sequences. The following criteria (see Materials and Methods for more details) were employed for defining a candidate coding-MULE. First, the element should contain a pair of TIRs and the distance between the two TIRs should be 2 to 30 kb (including nested TE insertions inside the element). Second, the sequence located within the TIRs should have homology (NCBI TBLASTN, e<10^−5^) to known transposase. Third, a TSD should be immediately flanking the TIRs of a single element. With these criteria, we detected 530 and 476 candidate coding-MULEs in maize and rice, respectively (Table S2A, S2B). Due to the presence of nested TE insertions in maize and rice [37]–[40], the candidate coding-MULEs were first masked using TE libraries and nested insertions were identified based on the output of RepeatMasker. After removing nested TE insertions, most candidate coding-MULEs (>95%) are between 2 to 10 kb in size in both maize and rice. However, the number of elements within different size ranges exhibits different patterns in the two species. In maize, a considerable portion of candidate coding-MULEs ranges from 2 to 3.5 kb (∼20%) while only 9% of the rice elements fall into the same category (χ^2^ = 25.7360, p<0.0001; Figure 2). The minimum length of known autonomous MULEs in plants is around 3.5 kb (Table S1), suggesting that elements smaller than 3.5 kb are unlikely to encode an intact transposase (transposase containing both DNA-binding and catalytic domains and no other coding defects). Most (71%) candidate coding-MULEs in rice are between 3.5 to 8 kb while only 58% of the elements in maize are within this range. As a result, candidate coding-MULEs with relatively small (2–3.5 kb) and large size (> 8 kb) are more prevalent in maize (42%) than that in rice (29%) (χ^2^ = 20.3473, p<0.0001)

To determine the phylogenetic relationship of these candidates, protein sequences containing the catalytic (DDE) domain within the elements were used to construct a phylogenetic tree. The phylogenetic analysis revealed that all elements belong to four distinct phylogenetic clades (Figure 1A). Clade I is represented by *MURA*, the first known MULE transposase [8]; and *TEDA*, a recently discovered MULE in maize [41]. Clade II is represented by *AtMU1*, an active element in *Arabidopsis* [24] and *Os3378*, an active MULE element in rice [31]. The representative element for clade III is *TRAP*, a MULE in maize [42]. The remainder of elements comprises clade IV, which is represented by *Jittery* and *FAR1*. *Jittery* is another active MULE from maize [26] and *FAR1* is a gene domesticated from a MULE transposase in *Arabidopsis* [27]. As shown in Figure 1A, clade I and IV contain more maize elements (green triangles) while rice elements (blue triangles) are more expanded in clade II and III.

### Nested insertions within the candidate coding-MULEs

**Figure 3:**
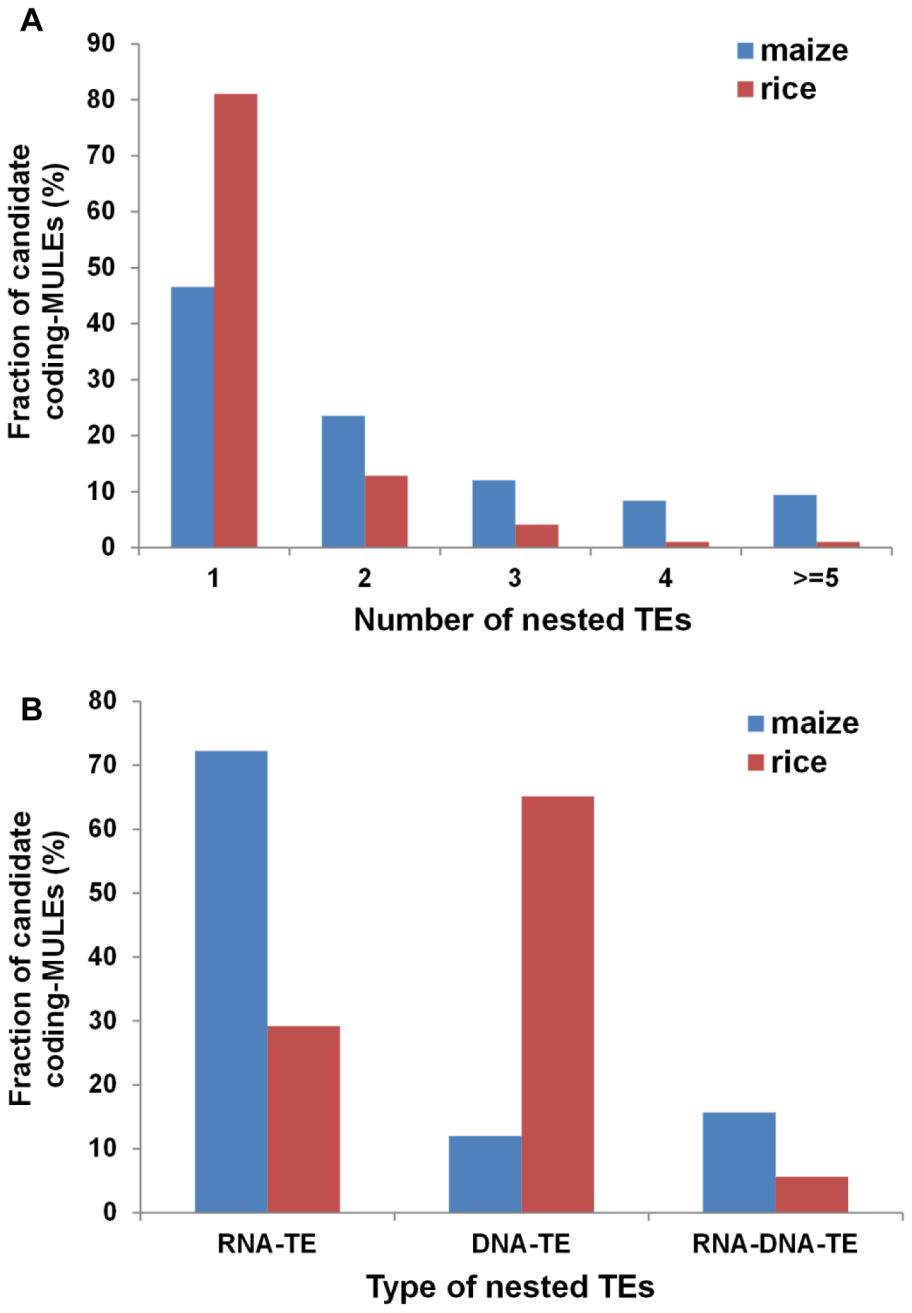
Fractions of candidate coding-MULEs containing different numbers (A) and types (B) of nested TE insertions.

As mentioned above, nested insertions of some TE families are very common in maize and rice [37]–[40]. If a TE inserts into a coding-MULE, it may interrupt the transposase ORF and abolish its function. To test whether this is the case, all TEs located within the candidate coding-MULEs were examined. The numbers of candidate coding-MULEs containing nested insertions are largely comparable between maize (n = 191, 36%) and rice (n = 195, 41%) (χ^2^ = 2.5761, p = 0.1085). However, among the 195 elements with nested insertions in rice, 107 of them are highly similar to each other, where 40 contain an intact Os0548 (a *Tourist*-like miniature inverted repeat transposable element, MITE, which is 274 bp in length) and 67 contain a partial Os0548 at the same site. As a result, the 107 elements are likely copies derived from a single insertion event where 40 of them are amplifications of the element with the intact Os0548 and meanwhile one of them may have experienced partial deletion of Os0548 followed by proliferation to 67 copies. Apparently, the insertion of this element did not abolish its capability for further transposition. In contrast, the 191 elements in maize all harbor independent insertions. If we exclude the 107 elements containing Os0548 from rice, the fraction of candidate coding MULEs with nested insertions is much lower in rice ([195–107] / [476–107] * 100 ≈ 24%) than that in maize (36%) (χ^2^ = 15.1021, p<0.0001). Moreover, the number and pattern of inserted TEs in individual candidate coding-MULE is different between maize and rice. Among the elements with nested insertions, most (∼81%) contain only a single TE insertion in rice whereas that in maize is only less than 47% (χ^2^ = 49.6349, p<0.0001) (Figure 3A). The fact that candidate coding-MULEs in maize contain more nested TEs than that in rice is also obvious when comparing the average number of nested insertions per coding-MULE with nested TEs (2 nested insertions in maize vs. 1 in rice).

Based on the class (see Introduction) of the inserted TEs, coding-MULEs with nested insertions were divided into three categories, *i.e.*, coding-MULEs with RNA-TE insertions (all inserted TEs were RNA TEs), coding-MULEs with DNA-TE insertions (all inserted TEs were DNA TEs), and those with RNA-DNA-TE insertions (with insertions from both RNA and DNA TEs). It turned out that over 72% of the candidate coding-MULEs in maize contained RNA-TE insertions compared with 29% of that in rice (χ^2^ = 71.4396, p<0.0001, Figure 3B). In contrast, there are more candidate coding-MULEs contain DNA-TE insertions in rice (65%) than that in maize (12%) (χ^2^ = 112.5264, p<0.0001). Furthermore, the average size of the inserted TEs in maize is about 3-fold of that in rice for DNA-TE (708 bp vs. 245 bp, p = 0.0016, t-test) and 1.3-fold for RNA-TE (6818 bp vs. 5081 bp, p = 0.0006, t-test) (Table 1). Accordingly, maize candidate coding-MULEs contain more independent and larger TE insertions, with most insertions from LTR retrotransposons. Interestingly, most maize MULEs (10 out of 14 vs. 26% of genome average, elements with purple dots in Figure 1A) grouping with *TRAP* (clade III) are associated with nested insertions of LTR retrotransposons while they are not particularly older or distribute differently than other MULEs. This suggests there might be some structural features of these elements that attract LTR elements.

**Table 1:** Number and average length of nested TE insertions based on TE classes in the candidate coding-MULEs.

	RNA-TE*	DNA-TE*	Total
Maize	329 (6818a†)	74 (708a)	403
Rice	99 (5081b)	152 (245b)	251

* Numbers in parenthesis represent the average length (bp) of nested TEs.

†Means in parenthesis in each column followed by different letters are significantly different (p<0.005, t-test).

**Figure 4:**
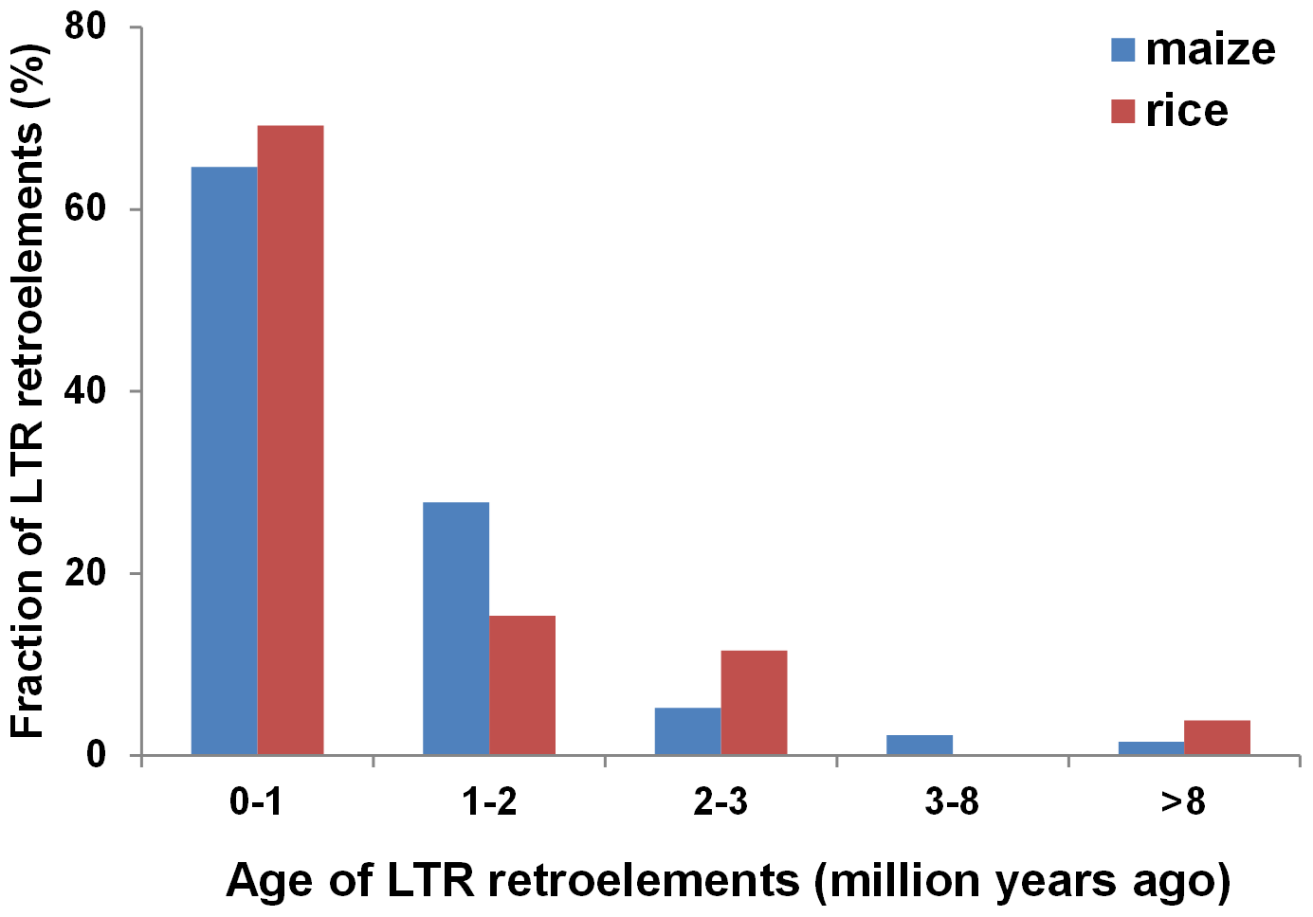
Ages of intact LTR retroelements in the candidate coding-MULEs in maize and rice.

To date the insertion time of the nested TEs, we analyzed all the intact LTR retrotransposons within the candidate coding-MULEs, which are characterized with long terminal repeat (LTR) at both ends of the element. The LTRs of one element are identical when inserted into a new location and become divergent over time. As a result, the identity between the LTRs has been used to estimate the age of the insertion [43]. In light of this, we calculated the approximate ages of 133 and 25 intact LTR elements within the candidate coding-MULEs in maize and rice, respectively (Figure 4). The largest fractions were estimated to be inserted within 1 million years in both maize (∼65%) and rice (∼69%), followed by insertions which have occurred 1 to 2 million years ago. Few LTR insertions were older than 3 million years in both genomes. Therefore, the insertion of LTR elements into the candidate coding-MULEs occurred within the same evolutionary time frame in both genomes despite the fewer insertions observed in rice.

As a comparison, we also examined how many coding-MULEs inserted into other TEs. It turned out that only 9% of the maize candidate elements and 7% of the rice elements inserted into other TEs (Table 2). This is in contrast to the fact that over 1/3 of the elements harbor insertions from other TEs, suggesting the coding-MULEs are more likely serving as targets for other elements rather than targeting other TEs. In addition, more maize candidate coding-MULEs (7%) inserted into RNA TEs than the rice elements (3%) (Table 2), which is consistent with the fact that there are many more RNA TEs in the maize genome than that in rice [1], [6].

**Table 2:** Insertion preference of candidate coding-MULEs (CMs).

	Maize			Rice		
Types of TEs	RNA-TE (% of total CMs*)	DNA-TE (% of total CMs*)	RNA/DNA	RNA-TE (% of total CMs*)	DNA-TE (% of total CMs*)	RNA/DNA
Nested TE insertion events in coding-MULEs	329 (32%)	74 (10%)	4.45	99 (19%†)	46 (9%†)	2.15
Coding-MULE insertion events in other TEs	35 (7%)	9 (2%)	3.89	15 (3%)	19 (4%)	0.79

* Note that individual coding-MULE may contain different number or types of nested TEs.

†In rice, 107 candidate coding-MULEs are copies of two elements resulting from one TE insertion event (see results), which was corrected in both the number of DNA-TE insertion events and the total candidate coding-MULEs (476–107+1 = 370).

### Coding capacity of the candidate coding-MULEs

Previous studies indicate that class II transposases, including MULE transposase, consist of an N-terminal helix-turn-helix (HTH) DNA-binding domain and a C-terminal DDE catalytic domain [22], [23], [44], [45]. The DNA binding domain is responsible for binding to the transposon DNA especially the TIR sequences and the catalytic domain is responsible for excision and integration of the element [34], [46]. To determine whether an individual candidate coding-MULE contains both domains, the coding regions of these elements were extracted from the relevant gene annotation or annotated manually. Subsequently, the presence of a DNA-binding domain was determined using the Jpred3 program and that of the catalytic domain by examination of a multiple sequence alignment containing *MURA* protein as the reference (see Materials and Methods for details). A transposase containing both DNA-binding and catalytic domains and without other defects (*e.g.*, frameshift, premature stop codon) in its ORF is considered a potentially intact transposase.

To obtain a better view, the candidate coding-MULEs were divided into six groups based on their coding capacity. Group 1 includes coding-MULEs with putative intact transposase, *i.e.*, at least one HTH domain (some elements have more than one HTH domain, *e.g.*, *Mos1* [34]) close to the amino terminus and a DDE catalytic domain close to the carboxyl terminus of the transposase protein. Group 2 is comprised of coding-MULEs with mutation of either translation start codon (ATG) or key amino acids (D, D, E) in the catalytic domain. Group 3 and 4 include coding-MULE transposases containing frameshift and premature stop codon, respectively. Those containing both frameshift and premature stop codon were assigned to Group 5. Group 6 consists of coding-MULEs with various forms of deletions in their transposases, including deletions of the DNA-binding domain and/or the catalytic domain or other regions. Since many candidate coding-MULEs contain several types of coding defects, one element may be assigned to more than one group (Table 3A). Meanwhile, a more specific classification where each element is only assigned to one sub-type is available in Table 3B.

**Table 3:** Candidate coding-MULEs (CMs) with or without distinct defects in their coding regions (redundant grouping), and candidate coding-MULEs (CMs) with or without distinct defects in their coding regions (non-redundant grouping).

A. Candidate coding-MULEs (CMs) with or without distinct defects in their coding regions (redundant grouping).								
Group	ORF* status		Maize			Rice		
			With TE (% of total CMs with nested TEs)	Without TE (% of total CMs without nested TEs)	Total	With TE (% of total CMs with nested TEs)	Without TE (% of total CMs without nested TEs)	Total
Group I	ORF with putative intact transposase		2 (1.05%)	29 (8.55%)	31 (5.85%)	70 (35.90%)	98 (34.88%)	168 (35.29%)
Group II	ORF with DDE or start codon mutation		19 (9.95%)	20 (5.90%)	39 (7.36%)	13 (6.67%)	14 (4.98%)	27 (5.67%)
Group III	ORF with frameshift		36 (18.85%)	37 (10.91%)	73 (13.77%)	34 (17.44%)	45 (16.01%)	79 (16.60%)
Group IV	ORF with premature stop codon		82 (42.93%)	91 (26.84%)	173 (32.64%)	64 (32.82%)	95 (33.81%)	159 (33.40%)
Group V	ORF with both frameshift and premature stop codon		19 (9.95%)	12 (3.54%)	31 (5.85%)	18 (9.23%)	33 (11.74%)	51 (10.71%)
Group VI	ORF with deletions		187 (97.91%)	288 (84.96%)	475 (89.62%)	66 (32.85%)	129 (45.91%)	195 (40.97%)
B. Candidate coding-MULEs (CMs) with or without distinct defects in their coding regions (non-redundant grouping).								
Group	ORF status		Maize			Rice		
			With TE (% of total CMs with nested TEs)	Without TE (% of total CMs without nested TEs)	Total	With TE (% of total CMs with nested TEs)	Without TE (% of total CMs without nested TEs)	Total
Group I	ORF with putative intact transposase		2 (1.05%)	29 (8.55%)	31 (5.85%)	70 (35.90%)	98 (34.88%)	168 (35.29%)
Group II	ORF with DDE or start codon mutation only		1 (0.52%)	4 (1.18%)	5 (0.94%)	3 (1.54%)	7 (2.49%)	10 (2.10%)
Group III	ORF with frameshift		1 (0.52%)	9 (2.65%)	10 (1.89%)	10 (5.13%)	4 (1.42%)	14 (2.94%)
Group IV	ORF with premature stop codon		0 (0%)	8 (2.36%)	8 (1.51%)	37 (18.97%)	28 (9.96%)	65 (13.66%)
Group V	ORF with both frameshift and premature stop codon		0 (0%)	1 (0.29%)	1 (0.19%)	9 (4.62%)	15 (5.34%)	24 (5.04%)
Group VI	ORF with deletions	deletion only	81 (42.41%)	185 (54.57%)	266 (50.19%)	37 (18.97%)	64 (22.78%)	101 (21.22%)
		with DDE or start codon mutation only	8 (4.19%)	5 (1.47%)	13 (2.45%)	5 (2.56%)	5 (1.78%)	10 (2.10%)
		with frameshift	16 (8.38%)	16 (4.72%)	32 (6.04%)	6 (3.08%)	8 (2.85%)	14 (2.94%)
		with premature stop codon	63 (32.98%)	71 (20.94%)	134 (25.28%)	9 (4.62%)	34 (12.10%)	43 (9.03%)
		with both frameshift and premature stop codon	19 (9.95%)	11 (3.24%)	30 (5.66%)	9 (4.62%)	18 (6.41%)	27 (5.67%)
Total			191	339	530	195	281	476

* ORF: open reading frame.

Overall, rice contains many more candidate coding-MULEs with putative intact transposase than maize (35.29% vs. 5.85%) (χ^2^ = 137.0185, p<0.0001; Table 3A), and deletion is likely the most important factor contributing to the difference. Most of the elements in maize (>89%) are associated with various deletions within the transposase compared to only 41% of the elements with deletions in rice. The presence of premature stop codon is the second most frequent defect, but the frequency is similar between maize (32.64%) and rice (33.40%) (χ^2^ = 0.0658, p = 0.7975; Table 3A). For elements with other defects (mutation of DDE motif or start codon, frameshift, and presence of both frameshift and premature stop codon), no dramatic difference was observed between the two species (Table 3A). In addition, many more maize elements (∼40%) contain more than one type of defect than that in rice (< 25%) (χ^2^ = 25.1084, p<0.0001).

**Figure 5:**
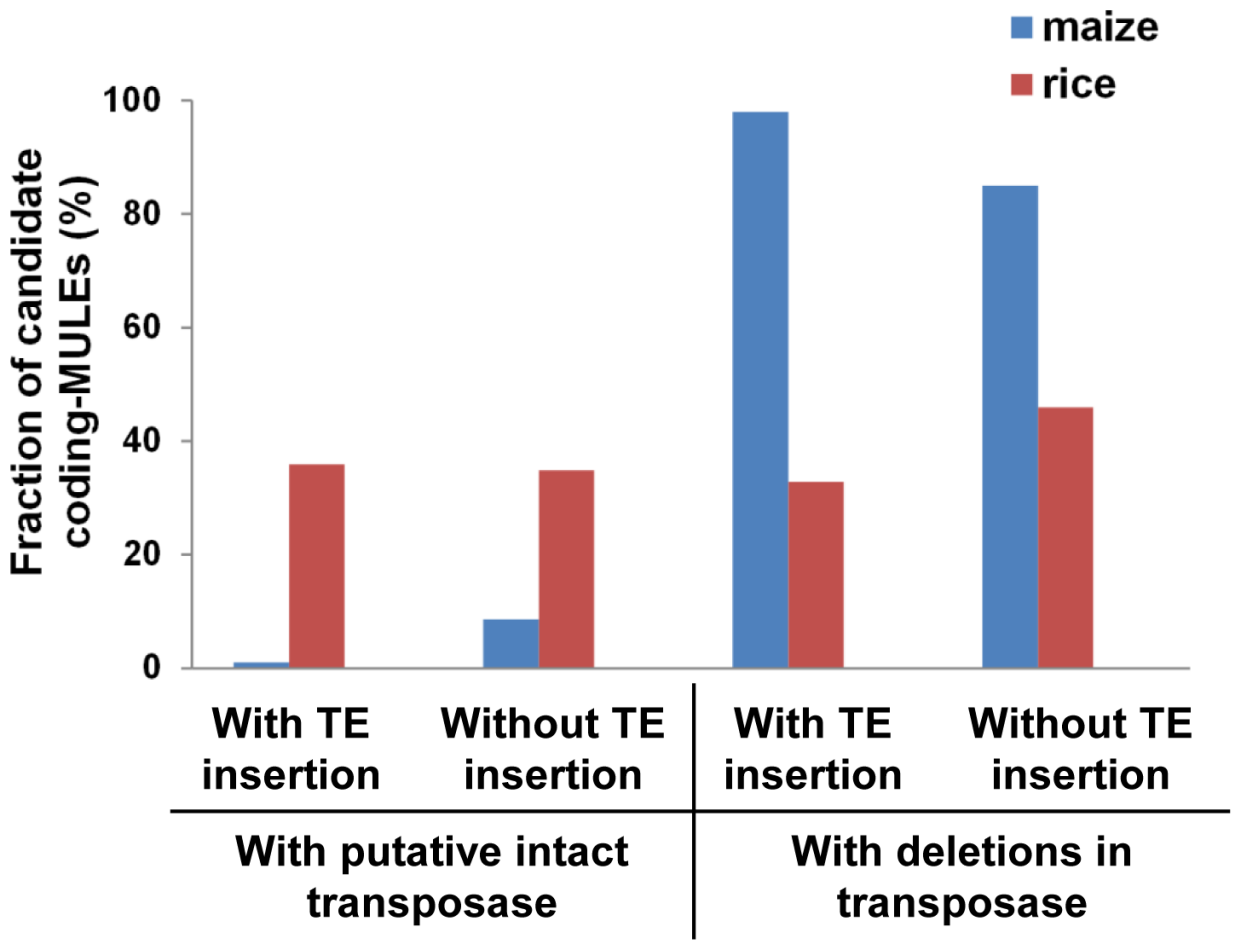
Fractions of candidate coding-MULEs containing putative intact transposases and transposases with deletions. The fractions were calculated within each category, *e.g.*, fraction of candidate coding-MULEs with TE insertions containing putative intact transposases is based on the total number of elements with TE insertions.

When classifying these elements based on whether they contain nested TE insertions or not, it is clear that in maize there are more candidate coding-MULEs containing putative intact transposase if there are no nested TEs (Figure 5). For example, only 2 out of 191 (1.05%) elements with nested insertions seem to have putative intact transposase, which is in contrast to 29 out of 339 (8.55%) elements without nested insertions in maize (χ^2^ = 12.5035, p = 0.0004; Table 3A). In contrast, ratios of candidate coding-MULEs harboring putative intact transposases for those with (35.90%) and without (34.88%) nested TE insertions (χ^2^ = 0.0526, p =  0.8185; Table 3A) are comparable in rice. Nevertheless, if the high copy elements (those containing intact and partial Os0548 element) were excluded, only 12.5% candidate coding-MULEs with nested insertions harbor putative intact transposase in rice, suggesting the destructive effect of nested TE insertions in both species, or elements with nested insertions are older than these without nested insertions so more mutations have accumulated. A close examination indicated that more nested TEs (n = 46) in maize directly disrupted transposases than that in rice (n = 23) (χ^2^ = 23.0278, p<0.0001), and most of them are LTR retrotransposons. Again, this indicates that nested TEs are more deleterious to coding-MULEs in maize than that in rice.

A second phylogenetic tree was constructed based on the catalytic domain of the elements with putative intact transposase (Figure 1B). Compared with Figure 1A, the number of elements in all the four clades declines, which is not surprising. This decrease is more obvious in maize, as revealed by the existence of only one clade (clade I) containing more than 10 elements and the other clades having four or less elements left. In contrast, rice (Nipponbare) still has more than 10 coding-MULEs in three clades (clade I, II, and III) and clade III seems to be active or recently active as evidenced by the short branch lengths (Figure 1B). In contrast, transposition activity in clades II to IV might be limited currently or in the future in maize (B73). This is consistent with the fact that there are either few maize elements (clades II and III) or many maize elements bear relatively long branches (clade IV) in these clades (Figure 1A), which are the signatures for loss of transposition activity.

### Other insertions and deletions within the candidate coding-MULEs

**Figure 6:**
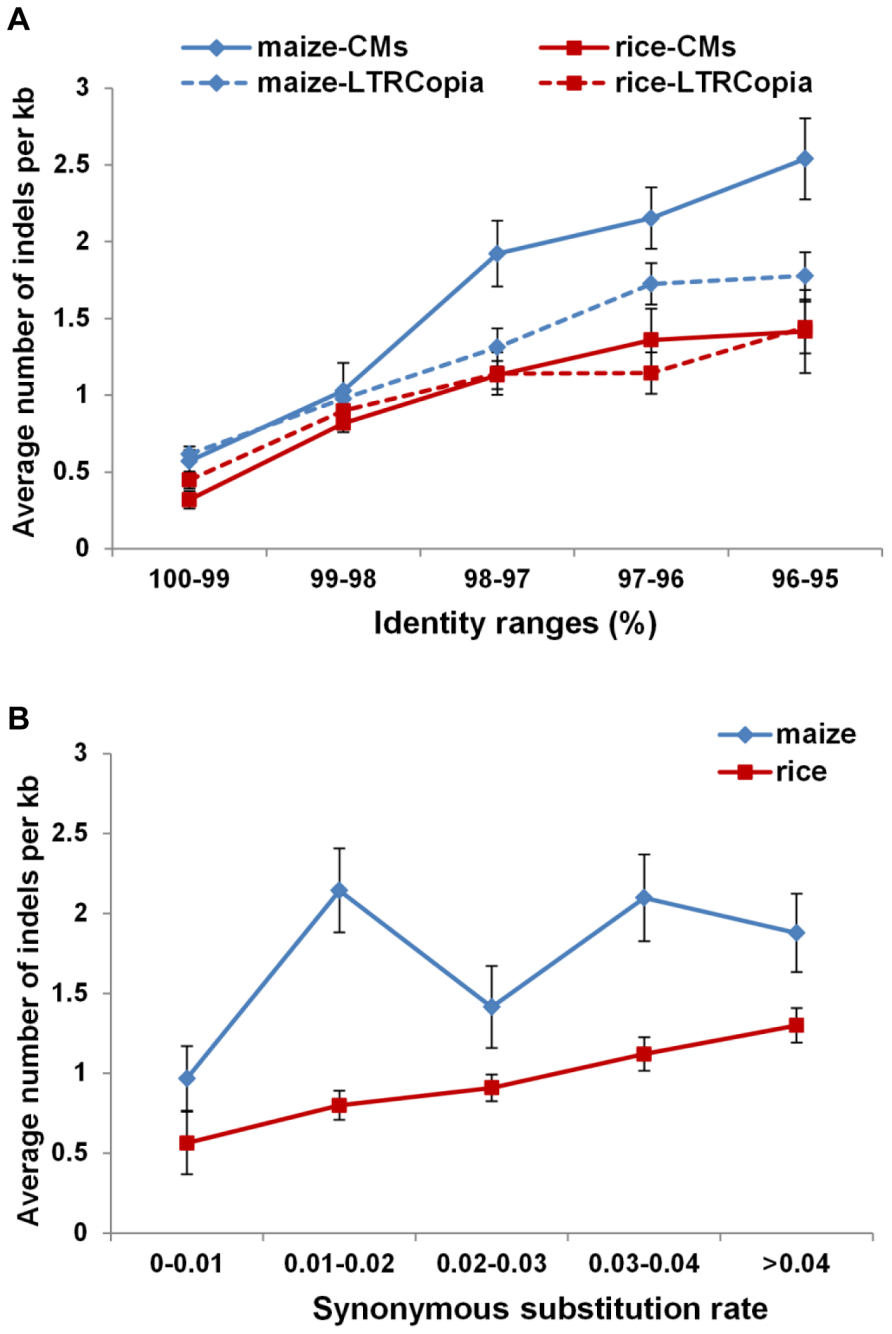
Average number of indels in maize and rice TEs. A). Average number of indels in candidate coding-MULEs (maize-CMs and rice-CMs) and *Copia*-like LTR elements using pairwise nucleotide identity as the grouping criterion; B). Average number of indels in candidate coding-MULEs using synonymous substitution rate as the grouping criterion.

The analysis above indicated that deletions in coding regions are prevalent in maize coding-MULEs, and this is possibly the most dominant factor for loss of coding capacity of these elements. The abundance of elements with deletions in maize could be attributed to a high deletion frequency in maize. Alternatively, the element with deletions may have an advantage in transposition (for instance, because of small size), so they have achieved relatively high copy numbers. To determine whether the frequency of insertion/deletion within the candidate coding-MULEs is similar between maize and rice, pairwise comparison was conducted using individual coding-MULEs and their most similar homologs. For accuracy, only pairs bearing higher than 95% identity in both maize and rice were considered. This portion of coding-MULE pairs were analyzed and the number and length of indels in those pairs were calculated and normalized to the average number of indels per kb (NIK) and the average length of indels per kb (LIK) (see Materials and Methods). The results show that the number of indels is significantly higher (p<0.05, t-test) in maize than that in rice in all the identity ranges except 98–99% (Figure 6A). The number of indels is increasing when the coding-MULE pair is less similar, with a ∼4.5 fold increase when the similarity is 95–96% compared to that of over 99% in both maize and rice. Meanwhile, the most significant difference between maize and rice is observed with the group containing element pairs with 95–96% identity. For this group, the NIK in maize is ∼2.48 and that in rice is ∼1.40, suggesting that there is at least one more indel per kb per coding-MULE pair generated in maize than those in rice. When comparing the LIK of coding-MULE pairs between maize and rice, no significant difference was observed and the average LIK values are 87.36 bp ± 16.06 for maize and 89.24 bp ± 11.21 for rice (p = 0.9235, t-test). To ensure the nucleotide identity reflect the divergence of homologous coding-MULE pairs, we also calculated indel rates based on synonymous substitution rates (Ks) between these pairs of elements in coding regions (Figure 6B), and a similar trend was observed. Taken together, it seems that coding-MULEs in maize experienced more indels than that in rice.

To determine whether the higher frequency of indels in maize is specific to coding-MULEs or a generic feature for the entire genome, we also conducted analysis of indels in LTR sequences of individual *Copia*-like LTR elements in the two genomes. Like that observed for coding-MULEs, maize LTR sequences seem to have experienced more frequent indels than that in rice. However, the difference is only evident when the nucleotide identity is within 96–97% (p = 0.0227, t-test). It should be noted that indel frequencies between LTRs and coding-MULEs in rice are not significantly different within the same identity range. In contrast, the average indel numbers in coding-MULEs are about two-fold of those in LTRs when the sequence identity ranges from 95% to 98% (Figure 6A). Collectively, these results demonstrate that maize may be more prone to incidence of indels than rice at the whole genome level. More importantly, within the maize genome, coding-MULEs seem to have experienced more indels than LTRs if we assume point mutation rate is comparable across different families of TEs.

### Expression evidence of the candidate coding-MULEs in maize and rice

A functional transposase is only available for transposition if the element is expressed. To determine how many candidate coding-MULEs are expressed, we searched the publically available EST and full-length cDNA (fl-cDNA) databases for evidence of expression. The completeness of these datasets was roughly assessed by determining the proportion of non-TE genes that have at least one match in the database with 99.5% or higher identity over the entire matched sequence. Under this criterion, we found 36% of non-TE genes in maize and 45% of that in rice having expression evidence, suggesting that the dataset for rice is 1.2-fold more comprehensive than that of maize. We did not exploit the data from next generation sequencing such as RNA-seq because most candidate coding-MULEs have highly similar copies in the genome and the short reads of RNA-seq experiments do not allow us to determine which specific element is expressed.

An element was considered to be expressed if it matches an fl-cDNA or EST sequence with at least 99.5% identity over the entire matched sequence (at least 300 bp). The results revealed that there were more coding-MULEs with either EST or fl-cDNA evidence in rice (n = 28) than that in maize (n = 9) (χ^2^ = 12.3933, p = 0.0004; Table 4A). Even if we take into account the 1.2-fold enrichment of expression evidence in rice than maize, rice still contains more elements (n = 23) (χ^2^ = 7.9969, p = 0.0047) with expression evidence. In both genomes, elements with expression evidence include coding-MULEs with and without nested TE insertions. In rice, expression evidence was detected for 4% (n = 7) of elements with putative intact transposase and 7% (n = 21) of elements with defects in transposases whereas those in maize are only 3% (n = 1) and 1.6% (n = 8), respectively (Table 4B). In either case, it does not appear that elements with intact transposases are more frequently transcribed. Interestingly, among the 7 elements containing putative intact transposases that are expressed in rice, three have a MITE insertion (among the 107 elements containing the Os0548 element) in the C-terminal region of the relevant coding-MULEs (∼2.5 kb downstream of the stop codon of the transposase ORF), which does not seem to affect the expression of the elements, at least at the transcription level (Table 4A). Overall, only a small subset of candidate coding-MULEs are expressed, yet rice has many more expressed elements than maize does, which may explain why the rice genome harbor many more MULEs. However, we must be cautious about interpreting this difference because it is known that expression of TEs is often induced under stress and the higher number of coding-MULEs expressed in rice may be due to the overrepresentation of stress conditions in the rice dataset.

**Table 4:** Candidate coding-MULEs with expression evidence in maize and rice, and summary of candidate coding-MULEs with expression evidence in maize and rice.

A. Candidate coding-MULEs with expression evidence in maize and rice.									
Species	Coding-MULE	With nested TE		EST/fl-cDNA*			ORF status		
Maize	CM-Zm373	Yes		BT069140			deletion only		
	CM-Zm391	Yes		gi|211046304|gb|FK977962.1|FK977962			deletion only		
	CM-Zm138	Yes		gi|211501343|gb|FL022227.1|FL022227			deletion only		
	CM-Zm216	Yes		gi|211515319|gb|FL470580.1|FL470580			deletion only		
	CM-Zm352	Yes		gi|211161841|gb|FK974923.1|FK974923			deletion & premature_stop_codon & frameshift		
	CM-Zm418	No		BT041283			deletion only		
	CM-Zm091	No		BT070085			deletion only		
	CM-Zm369	No		gi|211378400|gb|FL229430.1|FL229430			deletion only		
	CM-Zm137	No		gi|211249173|gb|FL479070.1|FL479070			putative intact		
Rice	CM-Os180	Yes		AK066496			deletion only		
	CM-Os087	Yes		gi|87004716|gb|CI306800.1|CI306800			deletion only		
	CM-Os392	Yes		gi|88668382|gb|CI721944.1|CI721944			deletion only		
	CM-Os222	Yes		gi|88692462|gb|CI736037.1|CI736037			deletion only		
	CM-Os464	Yes		gi|88846512|gb|CI373280.1|CI373280			deletion only		
	CM-Os133	Yes		AK072808			deletion & premature_stop_codon & frameshift		
	CM-Os028	Yes		AK288956			deletion & premature_stop_codon & frameshift		
	CM-Os046	Yes		AK120202			frameshift & premature_stop_codon		
	CM-Os181	Yes		AK067920			premature_stop_codon		
	CM-Os252	Yes		gi|88718219|gb|CI741729.1|CI741729			putative intact		
	CM-Os388	Yes		gi|88695069|gb|CI739107.1|CI739107			putative intact		
	CM-Os364	Yes		gi|88695796|gb|CI737373.1|CI737373			putative intact		
	CM-Os446	No		AK073736			deletion only		
	CM-Os006	No		gi|58687855|gb|CK076542.1|CK076542			deletion only		
	CM-Os083	No		gi|86437298|gb|CI119020.1|CI119020			deletion only		
	CM-Os089	No		gi|86827995|gb|CI274907.1|CI274907			deletion only		
	CM-Os422	No		gi|87030711|gb|CI361925.1|CI361925			deletion only		
	CM-Os209	No		gi|88276743|gb|CI397545.1|CI397545			deletion only		
	CM-Os317	No		gi|88727053|gb|CI750708.1|CI750708			deletion only		
	CM-Os217	No		AK066465			deletion & frameshift		
	CM-Os370	No		gi|88846132|gb|CI372900.1|CI372900			deletion & frameshift		
	CM-Os350	No		gi|88297974|gb|CI555989.1|CI555989			frameshift		
	CM-Os302	No		gi|29629482|gb|CB634491.1|CB634491			frameshift & premature_stop_codon		
	CM-Os018	No		gi|88730292|gb|CI753906.1|CI753906			premature_stop_codon		
	CM-Os395	No		gi|88279043|gb|CI400043.1|CI400043			putative intact		
	CM-Os200	No		gi|5701669|gb|C28952.2|C28952			putative intact		
	CM-Os044	No		AK100632			putative intact		
	CM-Os352	No		gi|29632126|gb|CB637135.1|CB637135			putative intact		
B. Summary of candidate coding-MULEs with expression evidence in maize and rice.									
	Coding-MULEs with putative intact transposase					Coding-MULEs with defects in transposase			
	With expression evidence		Without expression evidence		Total	With expression evidence		Without expression evidence	Total
Maize	1 (3.13%)		31 (96.88%)		32	8 (1.61%)		490 (98.39%)	498
Rice	7 (3.95%)		170 (96.05%)		177	21 (7.02%)		278 (92.98%)	299

* EST/fl-cDNA: expressed sequence tag/full length cDNA.

## Discussion

### Differential amplification of MULEs including coding-MULEs in rice and maize

The first active MULE element, *Mutator*, was found in maize due to the high mutation frequency it caused [8]. With the availability of a myriad of genome sequences, analysis of TEs has been carried out at the whole genome level, resulting in the discovery of the prevalence of MULEs in most living organisms [10]–[14], [25]. Being members of the grass family, both maize and rice contain thousands of MULEs [1], [15]. However, compared with rice (∼370 Mb available sequence), maize (∼2.1 Gb available sequence) contains less MULEs per unit genomic sequence (∼84 per Mb in rice vs. ∼8 per Mb in maize) as well as per unit coding sequence of non-TE genes (∼770/Mb in rice vs. ∼318/Mb in maize, based on maize genome annotation v2 and MSU rice annotation version 7). The persistence of TEs is an interaction between amplification through transposition, duplication of genomic sequences, and loss of TEs by excision and sequence erosion. Transposase, the protein encoded by autonomous elements, catalyzes transpositions, and is therefore responsible for increasing the copy number of the elements. In this study, we detected comparable numbers (530 vs. 476) of candidate coding-MULEs in maize and rice. Since all the candidate elements contain partial or complete transposases, they should have been derived from putative autonomous elements at a certain evolutionary stage. This implies that the resource for generating transposases was comparable between maize and rice in the traceable past, and the loss of such resource has been accelerated in maize in the recent past, which led to the reduced abundance of MULEs.

Maize and rice share an ancestor about 50–70 million years ago [30] and should have inherited largely the same set of transposable elements including MULEs. From this point of view, it is surprising that the phylogenetic composition of coding-MULEs is different between maize and rice, in addition to the difference in total copy numbers. The maize elements are abundant in clade I and IV, while the rice elements were amplified in clade II and III. This suggests that either the amplification of TEs is a rather fortuitous process or different genome environments may favor the survival of distinct elements.

### Factors involved in degeneration of coding-MULEs

In this study, we dissected various factors that may lead to the loss of coding capacity of MULE transposases. Apparently, for both maize and rice, deletion is the most devastating factor (Table 3A), which causes loss of the coding sequence or frameshift. Deletion of sequence is much more prevalent in maize than that in rice (90% vs. 40% of the elements). Point mutation is the second most important factor, which resulted in the mutation of DDE motif, the loss of a start codon, and the formation of premature stop codon. Nevertheless, the frequency of this type of mutation (∼40%) is comparable between maize and rice and therefore does not explain the differential amplification of MULEs between the two species. In addition, it is likely that point mutations within regions other than the DDE motif and the start codon may also lead to dysfunctional transposase. As a result, the number of functional transposases might be overestimated in this study. The third important factor is the insertion by other TEs, which may interrupt the ORF of the transposases or interrupt the *cis*-elements that are required for further transposition. Again, the number of elements that harbor nested insertions is comparable between maize and rice. However, the nested insertions in maize are more harmful than that in rice for a variety of reasons (see below). Thus, among the three major factors that demolish the coding capacity of MULEs, two of them are significantly enforced in maize.

Based on pairwise comparison of homologous elements, indels occur more frequently in maize than that in rice. Certainly, this is based on the assumption that the point mutation rate is comparable in the two species. Given the fact that number of elements with defects caused by point mutation is similar in maize and rice (see above), this is likely the case. In addition, our analysis using synonymous substitution rate led to similar results, further suggesting that functional constraint on nucleotide substitution is similar in the two species. Our analyses confirm previous studies of low stability of the maize genome [47]. By comparing an orthologous region of the maize, sorghum, and rice, Ilic *et al.* [48] found that the maize genome experienced more sequence deletions than rice, leading to the conclusion that the maize genome is less stable compared with the rice genome. The instability was partly attributed to the polyploidization event of maize. Several studies suggest that genome rearrangement was more frequent in species that experienced a polyploid event (reviewed in [49]). Unequal homologous recombination and illegitimate recombination are the two most proposed mechanisms responsible for DNA removal [50]. Woodhouse *et al.* [51] suggests that genes were preferentially deleted from one of the two maize homeologs possibly through a similar illegitimate recombination, which is also the primary source for TE removal in the maize genome.

The comparison of indel frequency between *Copia*-like LTR elements and coding-MULEs shed new lights on this issue. If we assume the frequency of point mutation is comparable across the two genomes, it is obvious that both types of elements demonstrate elevated indel frequency in maize, suggesting that in general indels occur more often in maize than that in rice. However, what is somehow unexpected is that coding-MULEs in maize seem to be subject to higher indel frequency than that of LTR elements and therefore contributes to their degeneration. Such difference could be attributed to the fact that they are located in different regions of the genomes and the indel rate is influenced by the recombination rate of the regions because it is known that TEs in different chromosomal domains evolve differently [52]. Alternatively, indels occur during transposition, so it could be due to the different transposition mechanism of different families of TEs as well. No matter what the underlying mechanism is, the difference in indel frequency explains, at least to some degree, why LTR elements are more successful than DNA TEs in maize.

In addition to the lower abundance of elements with putative intact transposase, the number of expressed candidate coding-MULEs in maize is only 1/3 of that in rice. The fewer expressed elements in maize is consistent with the low activity of MULEs in the genome, which is in contrast with the recent burst of amplification of LTR retrotransposons. It is well established that enormous variation of TE activities and compositions exist among different organisms (reviewed by [53], [54]). Some plant species, such as moss (*Physcomitrella patens*) and maize (*Zea mays*), exhibited relatively high LTR retrotransposon activity and low DNA TE activity while some animal species, *e.g.*, nematode (*Caenorhabditis elegans*) and brown bat (*Myotis lucifugus*), are more active in DNA TEs and less in LTR elements [54]. This suggests that genomes may have distinct mechanisms for silencing different types of elements that led to differential amplification of distinct TE families [53]. In fact, LTR elements, especially *gypsy*-like LTRs, express more frequently than DNA TEs in maize as revealed by more expressed sequence tags (ESTs) mapped to LTR retrotransposons than that to DNA TEs [55]. So far, it is unclear whether the lack of expression may further accelerate the degeneration of coding-MULEs in maize. For normal genes, those that are not expressed evolve more rapidly than genes that are highly expressed [56]–[59], and there is a correlation between expression level, Ka/Ks, and pseudogenization [60], [61]. As a result, it would not be surprising if the low expression frequency of the maize elements contributes to the loss of coding capacity of MULEs.

### Interaction between different TEs

Plant genomes harbor many distinct superfamilies of TEs and it is not known whether the amplification of some TEs impacts the amplification/survival of other TEs. Based on the genome-wide analyses of TEs, the rice genome harbors a total of 166,700 TEs [62] while that for maize is 1,283,000 [1]. If this is translated to TE insertions per unit sequence, the density of TEs in maize is 1.4-fold of that in rice (613 TEs/Mb sequence in maize vs. 439 TEs/Mb sequence in rice). From this point of view, the total number of nested TE insertions found in the candidate coding-MULEs (403 in maize vs. 251 in rice) is largely comparable to the genomic average and to each other in rice and maize. Nevertheless, nested TE insertions are more detrimental on the candidate coding-MULEs in maize than that in rice due to the following facts. First, the majority (∼88%) of the candidate coding-MULEs with TE insertions in maize contains RNA or retrotransposons (RNA-TE and DNA-RNA-TE), which are usually larger than DNA-TEs and are more disruptive (Figure 3B). Second, more candidate coding-MULEs in maize harbor two or more TE insertions (53%) compared with the majority of elements in rice that contain only one TE insertion (81%) (Figure 3A). This further increases the chance to abolish the functionality of the coding-MULEs, which is consistent with the fact that in maize, the proportion of elements with their transposases interrupted by nested TEs is twice of that in rice. Collectively, these nested TE insertions impaired a large fraction of coding-MULEs in maize, which is reflected by the presence of only two elements (∼1%) with potentially intact transposases among all the coding-MULEs containing nested insertions (Table 3A). On the other hand, few candidate coding-MULEs have inserted into other TEs including retrotransposons. This is likely because MULEs preferentially insert into low copy or genic sequences [63]. Such target specificity confers certain evolutionary advantages. For instance, the elements in genic regions are more likely to be expressed. Nevertheless, it may also bring about vulnerability to these elements when other elements such as retrotransposons are actively transposing. This is because retrotransposons tend to insert into other repetitive sequences so their activity is deleterious to other elements while the amplification of MULEs or other DNA transposons rarely interrupt retrotransposons. This is consistent with the results from a previous study where it was shown the incidents of insertions of other elements (including LTR elements) into miniature inverted repeat transposable elements (MITEs) were 65 times more often than that of MITEs into LTR and other DNA elements [40]. This indicates that MITEs are similar to coding-MULEs in terms of serving as targets for other elements rather than targeting other TEs.

Both theoretical modeling and empirical results demonstrated that mating systems play potential roles in shaping the abundance and diversity of transposable element within a genome [64]–[67]. A recent study showed that mode of reproduction contributed to the different transposon profiles in self-fertilizing *Arabidopsis thaliana* and its outcrossing relative *Arabidopsis lyrata* [65]. This study and other studies led to the conclusion that a reduced efficacy of natural selection against TE insertions in selfing populations [68]–[70]. Such reduced pressure against TE insertions provides more advantage for DNA TEs than RNA TEs because the insertion of DNA TEs are more likely in the genic regions which are subject to more intensive selection. Our comparison between rice (a selfing plant) and maize (an outcrossing plant) provides additional understanding about the possible influence of mating system on the dynamics of TEs. This is because outcrossing offers continuous opportunity for stochastic introduction of novel autonomous elements that may initialize new transposition activity. If a new retrotransposon is introduced, it is conceivable that the increased amplification of retrotransposons gradually abolishes the activity of MULEs or other DNA transposons through insertion into them. In contrast, the introduction of DNA TEs is not as harmful for retrotransposons. Taken together, the genomes of outcrossing plants are less favorable to DNA TEs due to the elevated efficacy of natural selection as well as the increased chances being attacked by retrotransposons. From this point of view, plants experiencing significant outcrossing are less likely to contain abundant DNA TEs and papaya is an excellent example.

## Supporting Information

Table S1 *MURA* and *MURA*-related transposases used in the study.
(DOC)

Table S2 A. Genomic coordinates and target site duplications (TSDs) of the candidate coding-MULEs in maize. B. Genomic coordinates and target site duplications (TSDs) of the candidate coding-MULEs in rice.
(DOC)

Table S3 Pairs of candidate coding-MULEs from maize (CM-Zm) and rice (CM-Os) used in calculating indels.
(DOC)

Data S1 (FAS)
