# Supplementary material for: Nested Insertions and Accumulation of Indels Are Negatively Correlated with Abundance of Mutator-Like Transposable Elements in Maize and Rice
Source: PLoS One. 2014 Jan 27;9(1):e87069. doi: 10.1371/journal.pone.0087069 (PMC3903597; doi:10.1371/journal.pone.0087069)
Supplement: Table S1 — MURA and MURA-related transposases used in the study. (DOC) [file pone.0087069.s001.doc]

Table S1. *MURA* and *MURA*-related transposases used in the study.

| Element | GenBank GI /Accession | Species | Length of putative Tpase (amino acids) | Element size (bp) | Reference |
| --- | --- | --- | --- | --- | --- |
| *Jittery* | 7673677/AAF66982 | *Zea mays* | 709 | 3916 | Xu et al., 2004 |
| *MURA* | 540581/AAA21566 | *Zea mays* | 823 | 4942 | Hershberger et al., 1991 |
| *TRAP* | 5690095/CAB51950 | *Zea mays* | 863 | 6393 | Comelli et al., 1999 |
| *AtMu1* | 2565011/AAB81881 | *Arabidopsis thaliana* | 761 | 3645 | Singer et al., 2001 |
| *Os3378* | 52353379/AAU43947 | *Oryza sativa* | 866 | 4394/4395 | Gao, 2012 |
| *FAR1* | 240255849/ NP_567455 | *Arabidopsis thaliana* | 827* | 4079* | Hudson et al., 2003 |
| *Hop* | 30421204/AAP31248 | *Fusarium oxysporum* | 836 | 3299 | Chalvet et al., 2003 |
| *Mutyl* | 50553866/XP_504344 | *Yarrowia lipolytica* | 1178 | 7413 | Neuveglise et al., 2005 |
| *RMUA* | 156723167/BAF79582 | *Oryza sativa* | 707 | 4374 | N/A^†^ |
| *MURA*-like | 194689672/ACF78920 | *Zea mays* | 601 | N/A | N/A |
| *MURA*-like | 223950329/ACN29248 | *Zea mays* | 751 | N/A | N/A |
| *MURA*-like | 12322384/AAG51216 | *Arabidopsis thaliana* | 826 | N/A | N/A |
| *MURA*-like | 5734742/AAD50007 | *Arabidopsis thaliana* | 622 | N/A | N/A |
| *MURA*-like | 7523705/AAF63144 | *Arabidopsis thaliana* | 726 | N/A | N/A |
| *MURA*-like | 17380908/AAL36266 | *Arabidopsis thaliana* | 749 | N/A | N/A |
| *MURA*-like | 41469647/AAS07370 | *Oryza sativa* | 747 | N/A | N/A |
| *MURA*-like | 22094356/AAM91883 | *Oryza sativa* | 896 | N/A | N/A |
| *MURA*-like | 15209152/AAK91885 | *Oryza sativa* | 959 | N/A | N/A |
| *MURA*-like | 29788811/AAP03357 | *Oryza sativa* | 907 | N/A | N/A |
| *MURA*-like | 51477400/AAU04773 | *Cucumis melo* | 807 | N/A | N/A |
| Hypothetical protein | 242096428/XP_002438704 | *Sorghum bicolor* | 720 | N/A | N/A |
| Predicted protein | 224122824/XP_002318925 | *Populus trichocarpa* | 580 | N/A | N/A |
| Hypothetical protein | 225432189/XP_002268620 | *Vitis vinifera* | 746 | N/A | N/A |

* *FAR1* has no identifiable terminal inverted repeats (TIRs), the length of putative transposase refers to the protein sequence of the gene and element size is the gene length.

† *MURA*-related transpsases with only NCBI depository and no detailed study are denoted as N/A for the element size and reference.

**References for Table S1**

Chalvet F, Grimaldi C, Kaper F, Langin T, Daboussi MJ (2003) Hop, an active *Mutator*-like element in the genome of the fungus *Fusarium oxysporum*. Molecular Biology and Evolution 20: 1362-1375

Comelli P, Konig J, Werr W (1999) Alternative splicing of two leading exons partitions promoter activity between the coding regions of the maize homeobox gene *Zmhox1a* and *Trap* (transposon-associated protein). Plant Molecular Biology 41: 615-625

Gao DY (2012) Identification of an active *Mutator*-like element (MULE) in rice (*Oryza sativa*). Molecular Genetics and Genomics 287: 261-271

Hershberger RJ, Warren CA, Walbot V (1991) *Mutator* activity in maize correlates with the presence and expression of the *Mu* transposable element *Mu9*. Proc Natl Acad Sci USA 88: 10198-10202

Hudson ME, Lisch DR, Quail PH (2003) The *FHY3* and *FAR1* genes encode transposase-related proteins involved in regulation of gene expression by the phytochrome A-signaling pathway. Plant Journal 34: 453-471

Neuveglise C, Chalvet F, Wincker P, Gaillardin C, Casaregola S (2005) *Mutator*-like element in the yeast *Yarrowia lipolytica* displays multiple alternative splicings. Eukaryotic Cell 4: 615-624

Singer T, Yordan C, Martienssen RA (2001) Robertson's *Mutator* transposons in *A. thaliana* are regulated by the chromatin-remodeling gene *Decrease in DNA Methylation* (*DDM1*). Genes & Development 15: 591-602

Xu ZN, Yan XH, Maurais S, Fu HH, O'Brien DG, Mottinger J, Dooner HK (2004) *Jittery*, a *Mutator* distant relative with a paradoxical mobile behavior: excision without reinsertion. Plant Cell 16: 1105-1114
