# Supplementary material for: Nested Insertions and Accumulation of Indels Are Negatively Correlated with Abundance of Mutator-Like Transposable Elements in Maize and Rice
Source: PLoS One. 2014 Jan 27;9(1):e87069. doi: 10.1371/journal.pone.0087069 (PMC3903597; doi:10.1371/journal.pone.0087069)
Supplement: Table S2 — A. Genomic coordinates and target site duplications (TSDs) of the candidate coding-MULEs in maize. B. Genomic coordinates and target site duplications (TSDs) of the candidate coding-MULEs in rice. (DOC) [file pone.0087069.s002.doc]

| Table S2A. Genomic coordinates and target site duplications (TSDs) of the candidate coding-MULEs in maize. | | | | | |
| --- | --- | --- | --- | --- | --- |
| Coding-MULE | Chromosome | Start | End | TSD-left | TSD-right |
| CM-Zm001 | chr1 | 111901612 | 111904903 | TCCGCCAGT | TCCGCTAGT |
| CM-Zm002 | chr1 | 116996419 | 117024336 | CGGCTCGC | CGGCTCGC |
| CM-Zm003 | chr1 | 126405575 | 126409774 | AAATATTAA | AAATATTTA |
| CM-Zm004 | chr1 | 154856955 | 154873843 | CAGTCGATT | CAATCGATT |
| CM-Zm005 | chr1 | 154958684 | 154962920 | TCTTGAAAG | TCTTGAAAG |
| CM-Zm006 | chr1 | 158556724 | 158565716 | TGCTATGGA | GCCTATGGA |
| CM-Zm007 | chr1 | 164399910 | 164408966 | GCCGCACCGT | GCCGCACCGT |
| CM-Zm008 | chr1 | 164926393 | 164940780 | CAGATATGA | CAGATATGA |
| CM-Zm009 | chr1 | 166893507 | 166902714 | ACATTCTAG | ACATTCTAG |
| CM-Zm010 | chr1 | 168466659 | 168476048 | TCGCTGATG | TCGCTGATG |
| CM-Zm011 | chr1 | 170719884 | 170784752 | ACTGACTGC | ACTAACTGC |
| CM-Zm012 | chr1 | 175251585 | 175291542 | TCCGGTACGT | TCCGGTACGT |
| CM-Zm013 | chr1 | 17718495 | 17723026 | CGCTGTTGG | CGCTGTTGG |
| CM-Zm014 | chr1 | 180391968 | 180394427 | GGTGATTC | GGTGATTC |
| CM-Zm015 | chr1 | 183469916 | 183476627 | ACCCACTTT | ACCCACTTT |
| CM-Zm016 | chr1 | 184601697 | 184610770 | GCAGCTCGA | GCAGCTCGA |
| CM-Zm017 | chr1 | 186044783 | 186053905 | TCTACGTGC | TCTACACGC |
| CM-Zm018 | chr1 | 186678966 | 186683056 | CTCGTCTTC | CTCGTCTTC |
| CM-Zm019 | chr1 | 193993497 | 193996657 | TTAGATTAC | TCAGATTAC |
| CM-Zm020 | chr1 | 197584741 | 197588823 | ACACTATACT | ACACTATACT |
| CM-Zm021 | chr1 | 198537108 | 198541168 | CAATATAT | CAATATAT |
| CM-Zm022 | chr1 | 206543934 | 206553108 | ATGCAGCAC | ACGCCGCAC |
| CM-Zm023 | chr1 | 212159243 | 212165307 | AGCTTCAGA | AGCTTCAGA |
| CM-Zm024 | chr1 | 216122847 | 216141008 | GTGTGGAGG | GTGTGGAGG |
| CM-Zm025 | chr1 | 216178647 | 216211284 | TTTACTATA | TTTACTATA |
| CM-Zm026 | chr1 | 217633844 | 217637832 | GAATCTGT | GAATCTGT |
| CM-Zm027 | chr1 | 21994328 | 22008911 | ACGACTCGC | ACGACTCGC |
| CM-Zm028 | chr1 | 222135977 | 222145087 | GCGCCGAGC | GCGCCGAGC |
| CM-Zm029 | chr1 | 223171995 | 223213579 | AGTAAATAA | AGTAAATAA |
| CM-Zm030 | chr1 | 226026529 | 226035245 | ACAGTCTGA | ACAGTCTGA |
| CM-Zm031 | chr1 | 230932209 | 230936230 | ATTCTTTTG | ATTCTTTTG |
| CM-Zm032 | chr1 | 232837999 | 232840505 | TCCATGAAA | TCTATGAAA |
| CM-Zm033 | chr1 | 237045923 | 237049140 | CTATTTTAA | CTATTTTAA |
| CM-Zm034 | chr1 | 2495983 | 2537264 | GGAGAATGA | GGAGAATGA |
| CM-Zm035 | chr1 | 253571516 | 253580714 | ACTCCACGC | ACTCCACGC |
| CM-Zm036 | chr1 | 258524102 | 258533881 | GCGCTTCAA | GCGCTTCAA |
| CM-Zm037 | chr1 | 259524032 | 259530854 | GTTTATCTG | GTTTATCTG |
| CM-Zm038 | chr1 | 263745020 | 263749282 | CATTAGAAA | CATTAGAAA |
| CM-Zm039 | chr1 | 265158328 | 265163132 | TTAGGTCGG | TTAGGTCGG |
| CM-Zm040 | chr1 | 265470307 | 265479470 | GCGCGACGC | GCGCGACGC |
| CM-Zm041 | chr1 | 265526973 | 265548195 | CGGTGGCCGC | CGGTGGCCGC |
| CM-Zm042 | chr1 | 27089066 | 27094491 | CTCCCAGCC | CTCCCAGCC |
| CM-Zm043 | chr1 | 272445753 | 272451847 | GCCAGGGA | GCCAGGGA |
| CM-Zm044 | chr1 | 273688174 | 273692422 | GTTTTTAG | TTTTTTAG |
| CM-Zm045 | chr1 | 276120570 | 276149871 | TTTGTAATT | TTTGTAATT |
| CM-Zm046 | chr1 | 277505793 | 277563159 | ACTAGTAAG | ACTAGTAAG |
| CM-Zm047 | chr1 | 277713046 | 277739072 | TGAGATGGC | AAAGATGGC |
| CM-Zm048 | chr1 | 279667809 | 279673464 | TCACCAACA | TCACCAACA |
| CM-Zm049 | chr1 | 281459097 | 281462980 | CAGAAGGG | CAGAAGGG |
| CM-Zm050 | chr1 | 28453520 | 28458732 | GTTGTAGTA | GTTGTAGTA |
| CM-Zm051 | chr1 | 288943778 | 288948637 | ACGTTAGGA | ACGTTAGGA |
| CM-Zm052 | chr1 | 29392689 | 29398024 | CTGTGTAGT | CTGTGTAGT |
| CM-Zm053 | chr1 | 293909607 | 293929203 | ACCGAGGGA | ACCGAGGGA |
| CM-Zm054 | chr1 | 34271943 | 34280945 | CGAAATGGA | CGAAATGGA |
| CM-Zm055 | chr1 | 34741398 | 34750275 | CAGGTGTG | CAGGTGTG |
| CM-Zm056 | chr1 | 35889167 | 35915341 | GTTAAGATG | GTTAAGATG |
| CM-Zm057 | chr1 | 37573470 | 37586490 | ACCGAATG | ACCGAATG |
| CM-Zm058 | chr1 | 39220065 | 39230882 | GGTCTTTAAG | GGTCTTTAAG |
| CM-Zm059 | chr1 | 40179762 | 40185515 | TCTCTGGTG | TCTCTGGTG |
| CM-Zm060 | chr1 | 45784824 | 45827466 | CCAGGCGGGT | CCTGGCGGG |
| CM-Zm061 | chr1 | 4877878 | 4897853 | ACGGGAGGA | ACGGGAGGA |
| CM-Zm062 | chr1 | 48642583 | 48648100 | GCCCGGGTG | GCCCGGGTG |
| CM-Zm063 | chr1 | 52821733 | 52827056 | GCAAGGTGG | GCAAGGTGG |
| CM-Zm064 | chr1 | 59390095 | 59462153 | CCGAATTAC | CCGAATTAC |
| CM-Zm065 | chr1 | 61595625 | 61612905 | GTTGAATCA | GTTGAATCA |
| CM-Zm066 | chr1 | 64711443 | 64716706 | TAATAAAAA | TAATAAAAA |
| CM-Zm067 | chr1 | 66799323 | 66803794 | TGGCCCGTG | TGGCCCGTG |
| CM-Zm068 | chr1 | 7049184 | 7054233 | ATTTTATAA | ATTTTATAA |
| CM-Zm069 | chr1 | 74279000 | 74281732 | GGCTGTTGC | GGTTGTTGC |
| CM-Zm070 | chr1 | 994277 | 997106 | GGATGAAACG | GGATGAAACG |
| CM-Zm071 | chr1 | 95102268 | 95105279 | CAACCAGACA | CAACTAGACG |
| CM-Zm072 | chr1 | 98503178 | 98542604 | GCCGGTATGT | GCCGGTATGT |
| CM-Zm073 | chr1 | 99985729 | 99989204 | TCGAGGTC | TCGAGGTC |
| CM-Zm121 | chr2 | 101465432 | 101475010 | AATGTGAGA | AATGGGAGA |
| CM-Zm122 | chr2 | 105033181 | 105038537 | TATAATTGG | TATAATTGG |
| CM-Zm123 | chr2 | 105464480 | 105485444 | CCTATTGGG | CCTATTGGG |
| CM-Zm124 | chr2 | 107878808 | 107887942 | TCAGCGTGA | TCAGCGTGA |
| CM-Zm125 | chr2 | 10882382 | 10885328 | GGGGAATCG | GGGGAATCG |
| CM-Zm126 | chr2 | 108667737 | 108675795 | CCCTGGGCGC | CCCCGGGCTC |
| CM-Zm127 | chr2 | 109442925 | 109479185 | TAAATCAAT | TAAATCTAT |
| CM-Zm128 | chr2 | 109782039 | 109804631 | CCATCGTAC | CCATCGTAC |
| CM-Zm129 | chr2 | 109870655 | 109876240 | CCCACCTCC | CCCACCTCC |
| CM-Zm130 | chr2 | 111893004 | 111913543 | TACGAATACG | TACGAATACG |
| CM-Zm131 | chr2 | 113960122 | 113966482 | ACGGCCACG | ACGGCCACG |
| CM-Zm132 | chr2 | 12908784 | 12918832 | TTCTGCTAG | TTCTGCTAG |
| CM-Zm133 | chr2 | 130525910 | 130529823 | AAAAAACTT | AAAAAACTT |
| CM-Zm134 | chr2 | 131752882 | 131756745 | ATCTGAGTG | ATCTGAGTG |
| CM-Zm135 | chr2 | 15283986 | 15288773 | CTGGCGCTC | CTGGCGCTC |
| CM-Zm136 | chr2 | 156194583 | 156197719 | GCCAATGAC | GCCAATGAC |
| CM-Zm137 | chr2 | 15876188 | 15880255 | GCGCGGAT | GCGCGGAT |
| CM-Zm138 | chr2 | 16000148 | 16021008 | TCTCTCTGC | TCTCTCTGC |
| CM-Zm139 | chr2 | 16948989 | 16951474 | ATCTTTTTGT | ATCTTTTTGT |
| CM-Zm140 | chr2 | 173586554 | 173603605 | GCGGTGGCA | ACGATGGCA |
| CM-Zm141 | chr2 | 176873952 | 176905648 | AAAAACATA | AAAAACATA |
| CM-Zm142 | chr2 | 178908899 | 178925055 | CCGCACATTG | CCGCACGTTG |
| CM-Zm143 | chr2 | 178872126 | 178875206 | CTCCACCGAG | CTCCACCGAG |
| CM-Zm144 | chr2 | 179040516 | 179056214 | GCTAAATAGC | GCTAAATAGC |
| CM-Zm145 | chr2 | 18004783 | 18029477 | CATAAGGCCC | CACAAGGCCA |
| CM-Zm146 | chr2 | 18135772 | 18142931 | CAGGCTCGC | CAGGCTCGC |
| CM-Zm147 | chr2 | 18158597 | 18202680 | ACAGACTCC | ACAAACCCC |
| CM-Zm148 | chr2 | 192749148 | 192751713 | CAGTAAAGC | CAGTAAAGC |
| CM-Zm149 | chr2 | 195164585 | 195169917 | AATCAAAAA | AATCAAAAA |
| CM-Zm150 | chr2 | 1979714 | 2017122 | GTTTCGAGAA | GTTTCGAGAA |
| CM-Zm151 | chr2 | 196957072 | 196961935 | GCCGGGGGC | GCCGGGGGC |
| CM-Zm152 | chr2 | 198446031 | 198450896 | TAATAAAAA | TAAAAAAAC |
| CM-Zm153 | chr2 | 199183379 | 199197242 | GCACCCAGG | GCACCCAGG |
| CM-Zm154 | chr2 | 200416579 | 200421813 | CCTTTCATG | CCTTTCATG |
| CM-Zm155 | chr2 | 200844637 | 200850990 | ACGGCCACG | ACGGCCACG |
| CM-Zm156 | chr2 | 20386823 | 20391100 | ATCCCCACCC | ATCCCCACCC |
| CM-Zm157 | chr2 | 203616895 | 203622734 | TCGCCTGTCC | TCTCCTGTCC |
| CM-Zm158 | chr2 | 204661678 | 204665202 | TATATAGAA | TATATAGAA |
| CM-Zm159 | chr2 | 209223934 | 209228080 | GATTTACGG | GATTTACGG |
| CM-Zm160 | chr2 | 210648487 | 210650733 | ACGGAACGG | ACGGAACGG |
| CM-Zm161 | chr2 | 210859297 | 210864597 | CTCTTCCC | CTCTTCCC |
| CM-Zm162 | chr2 | 211214271 | 211232307 | GTCTGGTGT | GTCTGATGT |
| CM-Zm163 | chr2 | 212728033 | 212743390 | CCAGCCGGT | CCAGCCGGT |
| CM-Zm164 | chr2 | 215136378 | 215143487 | CCTCACGGT | CCTCACGGT |
| CM-Zm165 | chr2 | 215261768 | 215281671 | TTCTTTTTT | TTCTTTTTT |
| CM-Zm166 | chr2 | 223489350 | 223495113 | CCCAGCAGCC | CCTAGCAGCC |
| CM-Zm167 | chr2 | 225499620 | 225508745 | TCTCGGGAG | TCCCGGGAG |
| CM-Zm168 | chr2 | 226959236 | 226962745 | AAAAAACAA | AAAAAACAA |
| CM-Zm169 | chr2 | 228822223 | 228831580 | CACCCGGGT | CACCCGGGT |
| CM-Zm170 | chr2 | 229682174 | 229700761 | TAGAATATA | TAGAATATA |
| CM-Zm171 | chr2 | 230525530 | 230532033 | GGCTTGGAG | GGCTTGGAG |
| CM-Zm172 | chr2 | 236241610 | 236244189 | CAGTTTTTT | CAGTTTTTT |
| CM-Zm173 | chr2 | 236434719 | 236439816 | CTCGTGCTGA | CTCGCGCTGC |
| CM-Zm174 | chr2 | 2440103 | 2444093 | GATCAGATG | GATCAGATG |
| CM-Zm175 | chr2 | 28897206 | 28927553 | TGGTTGACG | TGGTCGAAG |
| CM-Zm176 | chr2 | 32788540 | 32839963 | AGACCAGGA | AGACCAGGA |
| CM-Zm177 | chr2 | 34452442 | 34471497 | TAGATTTAA | TAGATTTAA |
| CM-Zm178 | chr2 | 3698857 | 3702080 | ACCAAATCAC | ACCAAATCAC |
| CM-Zm179 | chr2 | 36914325 | 36925133 | ATATTTTAATA | ATATTTTAATA |
| CM-Zm180 | chr2 | 42098505 | 42105079 | TGTCCACTG | TGTCCACTG |
| CM-Zm181 | chr2 | 43332446 | 43338095 | CCCACCGGG | CCCACCGAG |
| CM-Zm182 | chr2 | 45205783 | 45217629 | TTTAATTTA | TTTAATTTA |
| CM-Zm183 | chr2 | 4576209 | 4579876 | CCCTCCCCC | CCCTCCCCC |
| CM-Zm184 | chr2 | 45882682 | 45888992 | TCGAGCGAA | TCGAGCGAA |
| CM-Zm185 | chr2 | 4684500 | 4687506 | GATAAAAG | CATAAAAG |
| CM-Zm186 | chr2 | 47366052 | 47369794 | GTATAAATA | AAATAAATA |
| CM-Zm187 | chr2 | 52162051 | 52169644 | CCATAGTGG | CCATAGTGG |
| CM-Zm188 | chr2 | 55652797 | 55659385 | ACTCTTGCT | ACTCTTGCT |
| CM-Zm189 | chr2 | 58287805 | 58338968 | CAGCAAGCC | CAGCCAGCA |
| CM-Zm190 | chr2 | 69296801 | 69300583 | TTCGAGGGC | TTCGAGGGC |
| CM-Zm191 | chr2 | 76311477 | 76316246 | TCGAGAGGG | TCGAGAGGG |
| CM-Zm192 | chr2 | 86076633 | 86083822 | TTATTTCTA | TTATTTCTA |
| CM-Zm193 | chr2 | 86499397 | 86502906 | TTTTGTTCC | TTTTGTTCC |
| CM-Zm194 | chr2 | 88767150 | 88774977 | TAATTAAAA | TAATTAAAA |
| CM-Zm195 | chr2 | 9089157 | 9098005 | TCCATGTGC | TCCATGTGC |
| CM-Zm196 | chr2 | 92861844 | 92866081 | CCACCAAAAC | CCACCAAAAC |
| CM-Zm197 | chr2 | 93915403 | 93924437 | GCGACGTGG | GCGACGTGG |
| CM-Zm198 | chr3 | 107083417 | 107086961 | TCATCGTGC | TCATCGTGC |
| CM-Zm199 | chr3 | 109712745 | 109716909 | TACAAACAA | TACAAACAA |
| CM-Zm200 | chr3 | 127540035 | 127542112 | CATGCAGC | CATGCAGC |
| CM-Zm201 | chr3 | 131041236 | 131046825 | TAGAGGTAA | TAGAGGCAA |
| CM-Zm202 | chr3 | 135084126 | 135087609 | AGTCCTGCTG | AGTCCTGCTG |
| CM-Zm203 | chr3 | 135496949 | 135500432 | AACCCCGAC | AACCCTGAC |
| CM-Zm204 | chr3 | 135754189 | 135756416 | CATTATATG | CATTATATG |
| CM-Zm205 | chr3 | 137248111 | 137250950 | GGCCATGCT | GCCCATGCT |
| CM-Zm206 | chr3 | 141326020 | 141353535 | ATTATATTT | ATTATATTT |
| CM-Zm207 | chr3 | 146869519 | 146900506 | GGCTGGGCG | GGCTGGGCG |
| CM-Zm208 | chr3 | 146970103 | 146974918 | TTTGTTTTA | TTTGTTTTA |
| CM-Zm209 | chr3 | 149089883 | 149095130 | CGACCTTAG | CGACCTTAG |
| CM-Zm210 | chr3 | 152184505 | 152189515 | TACTATGAAC | TACTATGAAC |
| CM-Zm211 | chr3 | 152899402 | 152959408 | TTCTTATGT | TTCTTAGGT |
| CM-Zm212 | chr3 | 1606257 | 1610011 | CATGAACCC | CATGAACCC |
| CM-Zm213 | chr3 | 15755964 | 15783890 | CCAGCCTC | CCGGCCTC |
| CM-Zm214 | chr3 | 157605849 | 157647004 | GTCCCCAGC | GTCCCCAGC |
| CM-Zm215 | chr3 | 160184262 | 160193490 | TACTGCTGG | TACTGCTGG |
| CM-Zm216 | chr3 | 165480174 | 165552683 | TGGTTGATC | TGGTTGATC |
| CM-Zm217 | chr3 | 165705724 | 165722579 | GCCAAGGCC | GCCAAGGCC |
| CM-Zm218 | chr3 | 168014541 | 168018092 | GATACCGGG | GATACCGGG |
| CM-Zm219 | chr3 | 174149536 | 174152319 | GCTGCTGCT | GCTGCTGCT |
| CM-Zm220 | chr3 | 179383439 | 179392588 | GCTCAGCGT | GCTCAGCGT |
| CM-Zm221 | chr3 | 181125723 | 181131291 | GTCCTAGTC | GTCCTATTC |
| CM-Zm222 | chr3 | 183995040 | 183998330 | GGGAAAAACC | GGGAAAAACC |
| CM-Zm223 | chr3 | 184284796 | 184310551 | AAAGATGAA | AAAGATGAA |
| CM-Zm224 | chr3 | 19003970 | 19008348 | TATTTCTTC | TATTTCTTC |
| CM-Zm225 | chr3 | 193143221 | 193147204 | TAATGTATA | TAATGTAAG |
| CM-Zm226 | chr3 | 194206713 | 194228296 | CCTTGTGTG | CCTTGTGTG |
| CM-Zm227 | chr3 | 19464631 | 19469227 | TCGTCGATC | TCGTCGATC |
| CM-Zm228 | chr3 | 20068219 | 20072317 | ACGGCAGTC | ACGGCAGTC |
| CM-Zm229 | chr3 | 20165963 | 20173154 | ACTCACCGG | ACTCACCGG |
| CM-Zm230 | chr3 | 205215334 | 205224034 | ACGAAGATG | ACGAAGATG |
| CM-Zm231 | chr3 | 206410071 | 206414009 | TACACGCA | TACACGCA |
| CM-Zm232 | chr3 | 20705125 | 20714364 | TGTAGGGGA | TGTAGGGGA |
| CM-Zm233 | chr3 | 221548017 | 221557397 | ACGCTGCGC | ACGCTGCGC |
| CM-Zm234 | chr3 | 22283889 | 22297127 | TGTATTAAGC | TGTATTAAGC |
| CM-Zm235 | chr3 | 223299692 | 223305499 | CTTCCCTCG | CTTCCCTCG |
| CM-Zm236 | chr3 | 224234882 | 224238308 | TCCCAATCC | TCCCAATCC |
| CM-Zm237 | chr3 | 230256402 | 230277781 | GCGCAGGCG | GCGCAGGCG |
| CM-Zm238 | chr3 | 231365913 | 231369345 | TATTTTTGA | TATTTTTGA |
| CM-Zm239 | chr3 | 231707092 | 231710669 | CCCATGCAG | CCCCTGCAG |
| CM-Zm240 | chr3 | 25610150 | 25616394 | ACGACGGGG | GCGACGGGG |
| CM-Zm241 | chr3 | 32921770 | 32930588 | TGTTGCCGT | TGTTGCCGT |
| CM-Zm242 | chr3 | 33157955 | 33162967 | CCCACTGCC | CTCGCTGCC |
| CM-Zm243 | chr3 | 34782105 | 34815598 | AACTCGCGA | AACTGGCGA |
| CM-Zm244 | chr3 | 37676982 | 37696037 | TTCCATCCG | TTCCATCTA |
| CM-Zm245 | chr3 | 39205984 | 39210449 | ACGTGCGTG | ACGTGCGTG |
| CM-Zm246 | chr3 | 39981557 | 40017686 | TCTTACATTA | TCTTAAATTA |
| CM-Zm247 | chr3 | 40447757 | 40454813 | CCACCTCGAT | CCACCTCGAT |
| CM-Zm248 | chr3 | 45224617 | 45228826 | CATTTGCG | CATTTGCG |
| CM-Zm249 | chr3 | 48077912 | 48101304 | ACAATTACA | ACAATTACA |
| CM-Zm250 | chr3 | 50972929 | 51007882 | TCATAATTA | TCGTAATTA |
| CM-Zm251 | chr3 | 56070263 | 56078140 | CTAGATTTA | CTAGATTTA |
| CM-Zm252 | chr3 | 56828747 | 56833813 | TTGTAACCT | TTTCAACCT |
| CM-Zm253 | chr3 | 65715182 | 65717354 | GATGCCGG | GATGCCGG |
| CM-Zm254 | chr3 | 8741015 | 8746859 | TCCATTCGC | TCCATTCGC |
| CM-Zm255 | chr3 | 9416901 | 9420977 | ATTTCCCGT | ATTTCCCGT |
| CM-Zm256 | chr3 | 97008248 | 97011980 | CATGTGGC | CATGTGGC |
| CM-Zm257 | chr4 | 100335438 | 100344374 | CCCAACGGC | CCCAACGGC |
| CM-Zm258 | chr4 | 11015651 | 11018103 | TACATTTTT | TATATTTTT |
| CM-Zm259 | chr4 | 110759300 | 110763784 | ACAGAACCAT | ACAGAACTGT |
| CM-Zm260 | chr4 | 12148100 | 12170531 | GCGTTGGGA | GCGTTGGGA |
| CM-Zm261 | chr4 | 130909004 | 130918634 | CGGCTGGAC | CGGCTGGAC |
| CM-Zm262 | chr4 | 131027587 | 131031284 | GACAGGGAC | GACAGGGAC |
| CM-Zm263 | chr4 | 135975669 | 136002526 | ATCATGAAT | ATCATGGAT |
| CM-Zm264 | chr4 | 137048011 | 137051298 | AAATAACAT | AAATAACAT |
| CM-Zm265 | chr4 | 138160428 | 138164662 | GATTGTGAA | GATTGTGAA |
| CM-Zm266 | chr4 | 138513574 | 138519587 | GTGTCCTGC | GCGCCCTGC |
| CM-Zm267 | chr4 | 141268069 | 141270530 | GTTTGTCTTG | GTTTGTCTTG |
| CM-Zm268 | chr4 | 167404277 | 167421476 | GTACATATG | GTACATATG |
| CM-Zm269 | chr4 | 169512729 | 169537514 | CCGGGCCAC | CCGGGCCAC |
| CM-Zm270 | chr4 | 171791847 | 171796838 | GCGGGCGGA | GCGGGCGGA |
| CM-Zm271 | chr4 | 177314498 | 177321384 | CCCATCGG | CCCATCGG |
| CM-Zm272 | chr4 | 178599809 | 178612771 | CTTATGTTGG | CTTATGTTGG |
| CM-Zm273 | chr4 | 180047534 | 180052800 | CCATGTAGT | CCATGTAGT |
| CM-Zm274 | chr4 | 183924013 | 183927459 | GACGTGGAT | GACGTGGAT |
| CM-Zm275 | chr4 | 186822505 | 186825796 | GGCCTGGAC | GGCCTGGAC |
| CM-Zm276 | chr4 | 190665599 | 190671534 | GACCTGCCC | GACCTGCCC |
| CM-Zm277 | chr4 | 198047971 | 198072083 | GAGGGAGAG | GAGGGAGAG |
| CM-Zm278 | chr4 | 198126613 | 198129545 | ATACCAGAG | ATACCAGAG |
| CM-Zm279 | chr4 | 198394350 | 198401881 | TCCCAGTAA | TCCCAGTAA |
| CM-Zm280 | chr4 | 199010351 | 199027955 | GATAGGTGG | GATAGGTGG |
| CM-Zm281 | chr4 | 203226562 | 203229810 | GTCTCGTTG | GTCTCGTTG |
| CM-Zm282 | chr4 | 208100044 | 208143596 | CCCACCCG | CCCACCCG |
| CM-Zm283 | chr4 | 210537034 | 210539748 | TATGTTTAA | TATGTTTAA |
| CM-Zm284 | chr4 | 214896976 | 214902810 | GTCCAAGTC | GTCCAAGTC |
| CM-Zm285 | chr4 | 222714245 | 222720237 | CTTTCTTTT | CTTTCTTTT |
| CM-Zm286 | chr4 | 229024922 | 229027831 | AATTTAATC | AATGTAATC |
| CM-Zm287 | chr4 | 231613880 | 231619812 | TATTATTTA | TATTATTTA |
| CM-Zm288 | chr4 | 25993076 | 26002757 | GCCCAAATAT | GCCCAAATAT |
| CM-Zm289 | chr4 | 27995018 | 28004217 | TCCCTCTTC | TCCCTCTTC |
| CM-Zm290 | chr4 | 29614038 | 29623883 | GTGAGAACTT | GTGAGAACTT |
| CM-Zm291 | chr4 | 33414746 | 33417225 | GATTTATGTG | GATTTATGTG |
| CM-Zm292 | chr4 | 34025023 | 34028244 | TGGCGGTTG | TGGCGGTTG |
| CM-Zm293 | chr4 | 34875649 | 34900142 | TTTTCTTTT | TTTTCTTTT |
| CM-Zm294 | chr4 | 3557977 | 3561881 | CCGCAACC | CCGCAACC |
| CM-Zm295 | chr4 | 35552966 | 35574158 | GCAGTGGTAG | GCAGTGGAAG |
| CM-Zm296 | chr4 | 36861611 | 36865966 | GGAACGCAT | GGAACGCAT |
| CM-Zm297 | chr4 | 37260839 | 37263634 | ATCGAGAAC | ATCCAGAAC |
| CM-Zm298 | chr4 | 38062887 | 38067110 | TAAAAAAT | TAAAAAAT |
| CM-Zm299 | chr4 | 40359051 | 40383476 | TCGAGCGCGG | TCGAGCGCGG |
| CM-Zm300 | chr4 | 40612157 | 40617859 | CCTCGGAGC | CCCCAGAGC |
| CM-Zm301 | chr4 | 42749966 | 42759069 | ACTCACATC | ACTCACATC |
| CM-Zm302 | chr4 | 43593802 | 43601973 | ACGGGAGTA | ACGGGAGTA |
| CM-Zm303 | chr4 | 46156153 | 46165338 | CCGGCTGGC | CCGGCTGGC |
| CM-Zm304 | chr4 | 53954783 | 53957087 | TTCGGTTCGT | TTCGGTTCGT |
| CM-Zm305 | chr4 | 62385082 | 62391368 | CTTACAATG | CTTCCAACG |
| CM-Zm306 | chr4 | 62575773 | 62578448 | GGGGGGGGG | GGGGGGGGG |
| CM-Zm307 | chr4 | 6538362 | 6543975 | TTGCACGGG | TTGCACGGG |
| CM-Zm308 | chr4 | 68619314 | 68643309 | TTAAATATA | TTAAAATATA |
| CM-Zm309 | chr4 | 7156159 | 7165324 | TAGATTGGA | TAGATTGGA |
| CM-Zm310 | chr4 | 74915136 | 74918717 | GATCACTAG | GATCACTAG |
| CM-Zm311 | chr4 | 79917584 | 79924130 | GTCTGTCGT | GTCTGTCGT |
| CM-Zm312 | chr4 | 80976737 | 80980539 | GAGTACAGC | GAGTACAGC |
| CM-Zm313 | chr4 | 81361387 | 81364133 | GTCTAAGGA | GTCTAAGGA |
| CM-Zm314 | chr4 | 83188969 | 83195727 | TCGTGTGTC | TCGTGTGTC |
| CM-Zm315 | chr4 | 97675828 | 97724675 | TGTCCTCTA | TGTCCTCTA |
| CM-Zm316 | chr5 | 104860138 | 104876121 | TGGATAGGC | TGGATAGGC |
| CM-Zm317 | chr5 | 10580971 | 10583889 | TCCAGTTCA | TCCATTTCG |
| CM-Zm318 | chr5 | 106267353 | 106276456 | CCTCCCTGA | CCTCCCTGA |
| CM-Zm319 | chr5 | 10997218 | 11053060 | CACGCGCGGA | CACGCGCGGA |
| CM-Zm320 | chr5 | 120755343 | 120784553 | GGTTTTTT | GGTTTTTT |
| CM-Zm321 | chr5 | 137003371 | 137048584 | TTCTTGATA | TTCTTTATA |
| CM-Zm322 | chr5 | 14166651 | 14175772 | TCAGGGATAA | TCAGGATAAA |
| CM-Zm323 | chr5 | 146711943 | 146724045 | ACCTTCCCT | TCCCCTCCA |
| CM-Zm324 | chr5 | 150618091 | 150623443 | CCTCCGAGA | CCTCCGAGA |
| CM-Zm325 | chr5 | 15140419 | 15143128 | GTTTTTTTC | GTTTTTTC |
| CM-Zm326 | chr5 | 15438850 | 15441277 | CATGCCACC | CATGCCACC |
| CM-Zm327 | chr5 | 155901933 | 155906957 | GCCTTGGGC | GCCTTGGGC |
| CM-Zm328 | chr5 | 156377448 | 156437731 | CTATCGTGTC | CTACCGTGTT |
| CM-Zm329 | chr5 | 158354342 | 158363590 | ACGAGGCAA | ACGAGGCAA |
| CM-Zm330 | chr5 | 158520397 | 158534756 | CCACTATAC | CCACTATAC |
| CM-Zm331 | chr5 | 167598728 | 167602943 | TAGGCAATG | TAGGCAATG |
| CM-Zm332 | chr5 | 181635121 | 181639018 | GCACGCAAC | GCACGCAAC |
| CM-Zm333 | chr5 | 185870527 | 185897708 | GAATGTTT | GAATGTTT |
| CM-Zm334 | chr5 | 186764747 | 186769152 | CATGCACTTC | CATGCACTTC |
| CM-Zm335 | chr5 | 186842766 | 186848247 | TAAAAAACCA | TAGAAAACCA |
| CM-Zm336 | chr5 | 192842943 | 192846268 | GTTTGCTTA | GTTTGCTTA |
| CM-Zm337 | chr5 | 196295560 | 196298326 | TGGAGGAG | TGGAGGAG |
| CM-Zm338 | chr5 | 197837061 | 197840404 | ACCCAGGGA | ACCCAGAGA |
| CM-Zm339 | chr5 | 199360916 | 199364829 | ATTTGCAAT | ATTTGCAAT |
| CM-Zm340 | chr5 | 199724872 | 199731705 | GCGGCTCGA | GCGGCTCGA |
| CM-Zm341 | chr5 | 20129805 | 20133593 | CCTAGTTGG | CCTAGTTGG |
| CM-Zm342 | chr5 | 204880260 | 204894086 | TTCGTCGAC | TTCGTCGGC |
| CM-Zm343 | chr5 | 206195075 | 206202925 | CCGACTGGC | CCGACTGGC |
| CM-Zm344 | chr5 | 210479409 | 210486906 | CCCGCATTT | CCCGCATCC |
| CM-Zm345 | chr5 | 211599770 | 211603109 | CTGCGTCAC | CTGCGTCAC |
| CM-Zm346 | chr5 | 22345265 | 22354201 | GCGAGGAGC | GCGAGGAGC |
| CM-Zm347 | chr5 | 271845 | 276055 | TAACTTAAG | TAACTTAAG |
| CM-Zm348 | chr5 | 26270225 | 26272594 | GTTATTCAT | GTTACTTCAT |
| CM-Zm349 | chr5 | 26597564 | 26602765 | AGCCCAGGA | AGCCTAGGA |
| CM-Zm350 | chr5 | 30762638 | 30768890 | GGATAGAT | GGATAGAT |
| CM-Zm351 | chr5 | 32848709 | 32852715 | TTAAAAAAA | TTAAAAAAA |
| CM-Zm352 | chr5 | 38156899 | 38201574 | TATATTATT | TATATTATT |
| CM-Zm353 | chr5 | 43556876 | 43564009 | ACCCACTGG | ACCCACTGG |
| CM-Zm354 | chr5 | 46925827 | 46944129 | GAGAGATGG | GAGAGATGG |
| CM-Zm355 | chr5 | 51313095 | 51325266 | CAAGCGTAG | CAAGCGTAG |
| CM-Zm356 | chr5 | 54143824 | 54154863 | CCGGCGCGA | CCGGCGCGA |
| CM-Zm357 | chr5 | 56846860 | 56850114 | CCCAAAGTA | CCCAAAGTA |
| CM-Zm358 | chr5 | 5783319 | 5790583 | CCCTGGCTG | CCCTGGCTG |
| CM-Zm359 | chr5 | 60268927 | 60278118 | CCTCATCA | CCTCATCA |
| CM-Zm360 | chr5 | 62006618 | 62012709 | CTCTCCCTC | CTCTCCCTC |
| CM-Zm361 | chr5 | 63161369 | 63169820 | ACGCCGATG | ACGCCGATG |
| CM-Zm362 | chr5 | 71095449 | 71100418 | GTTTTTCGG | GTTTTTCGG |
| CM-Zm363 | chr5 | 74050030 | 74054119 | GTCAAAATC | GTCAAAATC |
| CM-Zm364 | chr5 | 74681530 | 74686527 | CATTACTTA | CGTTACTTA |
| CM-Zm365 | chr5 | 77534627 | 77569808 | ATACGGATG | AGACGGATG |
| CM-Zm366 | chr5 | 79510064 | 79529930 | TTTGGTGAT | TTTGGTGAT |
| CM-Zm367 | chr5 | 9121873 | 9135670 | GGTTAGGTA | GGTTAGGTA |
| CM-Zm368 | chr5 | 97630960 | 97633903 | TCCTGGCTC | TCCCACCTC |
| CM-Zm369 | chr5 | 9982288 | 9993510 | GCCCAGAGA | GCCCAGAGA |
| CM-Zm370 | chr6 | 104452225 | 104454728 | CTCTCGGTC | CTCTCCCTC |
| CM-Zm371 | chr6 | 105411436 | 105423270 | TCACTATTG | TCACTATTG |
| CM-Zm372 | chr6 | 106806668 | 106812107 | GCCGCCGCT | GTCGTCGCT |
| CM-Zm373 | chr6 | 109901974 | 109922711 | TGAAAATA | TGAAAATA |
| CM-Zm374 | chr6 | 115381744 | 115386398 | GCCGGCCGT | GCCGGCCGT |
| CM-Zm375 | chr6 | 115582845 | 115592068 | ACGAGAAGA | ACGAGAAAA |
| CM-Zm376 | chr6 | 118281503 | 118289760 | GTCCTTAAT | GTCCTTAAT |
| CM-Zm377 | chr6 | 119171056 | 119185631 | TCTTTTGAG | TCTTTTGAG |
| CM-Zm378 | chr6 | 121115302 | 121120493 | GTCGGGAAG | GTCGGGAAG |
| CM-Zm379 | chr6 | 122045077 | 122049357 | GGCGCTGAG | GGCGCTGAG |
| CM-Zm380 | chr6 | 122406778 | 122410229 | TTTCTATTT | TTTCTATTT |
| CM-Zm381 | chr6 | 122958702 | 122961699 | GCTCGGAGA | GCTCGGAGA |
| CM-Zm382 | chr6 | 124881442 | 124890219 | ACGTCTAGG | ACGTCTAGG |
| CM-Zm383 | chr6 | 132240006 | 132247934 | TTAGCTTTC | TTAGCTTTC |
| CM-Zm384 | chr6 | 141219776 | 141225300 | GATATAATAA | GATATAATAA |
| CM-Zm385 | chr6 | 146490037 | 146497785 | TCGTACATG | TCGTACAGG |
| CM-Zm386 | chr6 | 147360019 | 147379549 | TGGAATGGA | TGGAATGGA |
| CM-Zm387 | chr6 | 148305579 | 148331861 | CCGGAGCGT | CCGGAGCGT |
| CM-Zm388 | chr6 | 149081974 | 149085543 | CAAATAGAA | CAAATAGAA |
| CM-Zm389 | chr6 | 14939165 | 14942588 | CCTTTGGAGGG | CCTTTGGAGGG |
| CM-Zm390 | chr6 | 152321575 | 152324571 | AGCTGGAAC | AGCTGGTTC |
| CM-Zm391 | chr6 | 15504872 | 15522402 | ATGGAAATT | ATGGAAATT |
| CM-Zm392 | chr6 | 155071024 | 155074330 | CTTTATAGT | CTTTATAGT |
| CM-Zm394 | chr6 | 160339166 | 160345768 | CTCGTGGGA | CTCCTGGGA |
| CM-Zm395 | chr6 | 161650423 | 161655481 | CTGGTAGTG | CTGGTAGTG |
| CM-Zm396 | chr6 | 163756071 | 163765345 | TTTTAAAAA | TTTTAAAAA |
| CM-Zm397 | chr6 | 163933305 | 163948454 | CCGACCGGC | CCGACCGGC |
| CM-Zm398 | chr6 | 164778090 | 164783386 | TAGCAGCGA | TAGCAGCGA |
| CM-Zm399 | chr6 | 16661184 | 16669062 | TTATTATTAC | TTATTATTAC |
| CM-Zm400 | chr6 | 168691689 | 168702060 | GCCAGAGGA | GCCAGAGGA |
| CM-Zm401 | chr6 | 168975559 | 168978884 | GGTTGGTTG | GGTTGGTTG |
| CM-Zm402 | chr6 | 24626511 | 24633368 | CCCCGCCTC | CCCCGCCTC |
| CM-Zm403 | chr6 | 49053290 | 49100725 | TCCCTGCGT | TCCCTGCGT |
| CM-Zm404 | chr6 | 65916800 | 65920240 | CCTCACGCC | CCTCACGCC |
| CM-Zm405 | chr6 | 69149694 | 69203359 | AAAATCAAA | AAAATCAAA |
| CM-Zm406 | chr6 | 69631106 | 69640574 | GTTGTTCGT | GTTGTTCGT |
| CM-Zm407 | chr6 | 82920120 | 82955897 | GGTTTGTTA | GCTTTGTTA |
| CM-Zm408 | chr6 | 86500513 | 86504109 | CATGCGAGG | CATGCGAGG |
| CM-Zm409 | chr6 | 89889026 | 89918075 | GGTGCGGCC | GGTGCGGCC |
| CM-Zm410 | chr6 | 93160560 | 93164656 | ATGCAGATG | GTGCGGATG |
| CM-Zm411 | chr6 | 93739203 | 93754358 | GTGTTGCAGC | GTGTCGCAGC |
| CM-Zm412 | chr6 | 93840377 | 93843253 | CAGCACGCG | CAGCACGCG |
| CM-Zm413 | chr6 | 94427252 | 94434923 | GCGCTATCCTA | GCGCTATCCTA |
| CM-Zm414 | chr6 | 95117749 | 95127738 | CAAGGAAGC | CAAGGAAGC |
| CM-Zm415 | chr6 | 96525984 | 96535035 | GCGTCGTGC | GCATCGTGC |
| CM-Zm416 | chr6 | 97624683 | 97631064 | CCAAACCGC | CCAAACCGC |
| CM-Zm417 | chr7 | 10144188 | 10156228 | GTTCCCGT | GTTCCCGT |
| CM-Zm418 | chr7 | 101894098 | 101901359 | TCACCTAGA | TCACCTAGA |
| CM-Zm419 | chr7 | 115020225 | 115023744 | GCGCTCGAA | TAGCTCGAA |
| CM-Zm420 | chr7 | 118086197 | 118090078 | AAAAACAAA | AAAAACAAA |
| CM-Zm421 | chr7 | 121242455 | 121248997 | GTCGTCGAG | GTCGTCGAG |
| CM-Zm422 | chr7 | 121271552 | 121278444 | CCTCGCCGA | CCTCGCCGA |
| CM-Zm423 | chr7 | 12338640 | 12346693 | ACGCGCCGC | ACGCGCCGC |
| CM-Zm424 | chr7 | 126056279 | 126059501 | ATCATCAAA | ATCATCAAA |
| CM-Zm425 | chr7 | 128565554 | 128567912 | TAAAATTA | TAAAATTA |
| CM-Zm426 | chr7 | 130105037 | 130129995 | CAAGGATA | CCAGGACA |
| CM-Zm427 | chr7 | 132633268 | 132639787 | CACGGCGTC | CACGGCGTC |
| CM-Zm428 | chr7 | 133716705 | 133733760 | TCAAAGAAA | TCAAAGAAA |
| CM-Zm429 | chr7 | 138901315 | 138909474 | GACGGCTGT | GACGGCTGT |
| CM-Zm430 | chr7 | 144220211 | 144228097 | AGCTGCCGGA | AGCTGCGGGA |
| CM-Zm431 | chr7 | 148687569 | 148699499 | TTTATAAAA | TTTATAAAA |
| CM-Zm432 | chr7 | 148898561 | 148903174 | TCGTTGCTA | TCGTCACTA |
| CM-Zm433 | chr7 | 148997050 | 149003422 | CGTCAGCG | CGTCAGCG |
| CM-Zm434 | chr7 | 152244504 | 152246511 | CACTATCGTG | CACTATCATG |
| CM-Zm435 | chr7 | 15545462 | 15548991 | TGAGAGTA | TGAGAGTA |
| CM-Zm436 | chr7 | 155020429 | 155023908 | TCAGTCCTA | TCAGTCCTA |
| CM-Zm437 | chr7 | 159773337 | 159777024 | CCAATTATT | CCAATTATT |
| CM-Zm438 | chr7 | 160481849 | 160508643 | ACGGCTGGG | ACGGCTGGG |
| CM-Zm439 | chr7 | 165192404 | 165195554 | GTGCGTAGG | GTGCGTAGG |
| CM-Zm440 | chr7 | 172080684 | 172084234 | GCGTGGCGA | GCGTGGCGA |
| CM-Zm441 | chr7 | 173300928 | 173319796 | GTAGCAGTC | GTAGCAGTC |
| CM-Zm442 | chr7 | 173661230 | 173670319 | TCTTATACA | TCTTATACA |
| CM-Zm443 | chr7 | 26600054 | 26603595 | GACTACATT | GACTACATT |
| CM-Zm444 | chr7 | 27596446 | 27604343 | CCGTGGCGG | CCATGGCGG |
| CM-Zm445 | chr7 | 31461442 | 31465571 | CTGTGAGTG | CTGTGAGTG |
| CM-Zm446 | chr7 | 48040103 | 48043019 | GCCCGCGAG | GCCCGCGAG |
| CM-Zm447 | chr7 | 72714306 | 72716922 | ACTAGGAGG | ACTAGGAGG |
| CM-Zm448 | chr7 | 78365911 | 78372601 | GACAGAGGC | GACAGAGGC |
| CM-Zm449 | chr7 | 7971150 | 8005375 | GATTTGGC | GATTTGGC |
| CM-Zm450 | chr7 | 87824650 | 87857047 | TATTATTGCA | TATTACTGCA |
| CM-Zm451 | chr7 | 89993663 | 89997370 | AAATTCTAG | AAATTCTAG |
| CM-Zm452 | chr7 | 9270815 | 9279969 | TACTGATGA | TACTGATGA |
| CM-Zm453 | chr8 | 10804776 | 10808953 | CGAGGGGAC | CGAGGGGAC |
| CM-Zm454 | chr8 | 109380622 | 109390502 | TACGTGGTA | TACGTGGTA |
| CM-Zm455 | chr8 | 117849818 | 117862746 | CCTGGGCTC | CCTGGGCTC |
| CM-Zm456 | chr8 | 122657126 | 122696421 | GCCTACTGG | GCCTACTGG |
| CM-Zm457 | chr8 | 122754864 | 122762214 | CACTTTTGAG | CACTTTTGAG |
| CM-Zm458 | chr8 | 124277430 | 124282180 | TATTTTACCG | TATTTTACCG |
| CM-Zm459 | chr8 | 12552062 | 12555081 | GTAGTAAAC | GTAGTAAAC |
| CM-Zm460 | chr8 | 125906632 | 125919991 | CATGTGTGC | CATGTGTGC |
| CM-Zm461 | chr8 | 126880576 | 126883996 | TTGATTTTT | TTGATTTTT |
| CM-Zm462 | chr8 | 128726519 | 128744481 | CTAGAACG | CTAGAACG |
| CM-Zm463 | chr8 | 132288444 | 132291667 | TTTCTCAG | TTTCTCAG |
| CM-Zm464 | chr8 | 133305615 | 133311843 | CTCCGACCA | CTCCGACCA |
| CM-Zm465 | chr8 | 13557165 | 13563622 | ACACTATTG | ACACTATTG |
| CM-Zm466 | chr8 | 144136889 | 144141403 | AAAAAGCTA | AAAAAACTA |
| CM-Zm467 | chr8 | 144533337 | 144537092 | GCCTGGCC | GCCTGGCC |
| CM-Zm468 | chr8 | 144605936 | 144633540 | CCCAGCGCG | CCCAGCGCG |
| CM-Zm469 | chr8 | 147513601 | 147522536 | ACATATCGA | ACATATCTA |
| CM-Zm470 | chr8 | 151036178 | 151058358 | GCACGCACGGC | GCACGCACAGT |
| CM-Zm471 | chr8 | 152706783 | 152713650 | GTCTCTAAC | GTCTCTAAC |
| CM-Zm472 | chr8 | 154472550 | 154478274 | CCGCCTGGT | CCGCCTGGT |
| CM-Zm473 | chr8 | 158123331 | 158126606 | AAAAAAAAA | AAAAAAAAA |
| CM-Zm474 | chr8 | 158562737 | 158565793 | TGATTTTTA | TGATTTTTA |
| CM-Zm475 | chr8 | 161127559 | 161132263 | CACCAGCA | CACCAGCA |
| CM-Zm476 | chr8 | 163315008 | 163365781 | TGAGCAGCGA | TGAGCAGCGA |
| CM-Zm477 | chr8 | 164565773 | 164574908 | GTAGAGGCGC | GTAGAGGCGC |
| CM-Zm478 | chr8 | 165295777 | 165299991 | TTTTATAA | TTTTATAA |
| CM-Zm479 | chr8 | 166748203 | 166777434 | CCTACGTGA | CCACCGTGA |
| CM-Zm480 | chr8 | 171419360 | 171428775 | AAAGGACAC | AAAGGACAC |
| CM-Zm481 | chr8 | 171996387 | 172000535 | CGTGCACATA | CGTGCACATA |
| CM-Zm482 | chr8 | 172870118 | 172872445 | TTAAAACA | TTAAAACA |
| CM-Zm483 | chr8 | 20928350 | 20953632 | GCTAGGGAG | GCTATGGAG |
| CM-Zm484 | chr8 | 21668298 | 21694627 | CCCATGGAA | CACATGGAA |
| CM-Zm485 | chr8 | 23640691 | 23660684 | CACCAGATC | CACCAGATC |
| CM-Zm486 | chr8 | 29874115 | 29876760 | GTATTGCT | GTATTGCT |
| CM-Zm487 | chr8 | 36006227 | 36012892 | AAAAAAAA | AAAAAAAA |
| CM-Zm488 | chr8 | 40804030 | 40826720 | TGTTTGAAT | TGTTTGAAT |
| CM-Zm489 | chr8 | 47428659 | 47431536 | TGCAGTACA | TGCAGTACA |
| CM-Zm490 | chr8 | 49848921 | 49853709 | GCCCAGATC | GCCCAGATC |
| CM-Zm491 | chr8 | 63240687 | 63267309 | CTAAAAAAAG | CTAAAAAG |
| CM-Zm492 | chr8 | 65092642 | 65096247 | AGATTTATA | AGATTTATA |
| CM-Zm493 | chr8 | 65722602 | 65749676 | TTTGCCTTAA | TTTGCCTTAA |
| CM-Zm494 | chr8 | 72763041 | 72772886 | TCTACGGCA | TCTACGGCA |
| CM-Zm495 | chr8 | 75040621 | 75055724 | GCAAAGCTA | GCAAAGCTA |
| CM-Zm496 | chr8 | 7999509 | 8003726 | CCTTCCCAAG | CCTTCCCAAG |
| CM-Zm497 | chr8 | 83728652 | 83733936 | CACAATTGC | CACAATTGC |
| CM-Zm498 | chr8 | 84259186 | 84264402 | GACGTTGGG | GACGTTGGG |
| CM-Zm499 | chr8 | 85150307 | 85159471 | TCACTGCTA | TCACTGCTA |
| CM-Zm500 | chr8 | 8864324 | 8874163 | TCGCGCGTG | TCGCGCGTG |
| CM-Zm501 | chr8 | 91313681 | 91319597 | TAGTTTAAT | TAGTTTAAT |
| CM-Zm502 | chr8 | 98983211 | 98992352 | TCAGACATG | TCAGACATG |
| CM-Zm503 | chr9 | 106073447 | 106076991 | CCCGCCCTC | CCCGCCCTC |
| CM-Zm504 | chr9 | 113795726 | 113801671 | CCTGCCTCA | CCTCCCTCA |
| CM-Zm505 | chr9 | 11764280 | 11774504 | GGGTTGGG | GGGCTGGG |
| CM-Zm506 | chr9 | 135722889 | 135728893 | AAAAACCA | AAAAACCA |
| CM-Zm507 | chr9 | 135818504 | 135833696 | AGAAGATGCA | AGAAGATGCA |
| CM-Zm508 | chr9 | 136380156 | 136389187 | ACGGCCTG | ACGGCCTG |
| CM-Zm509 | chr9 | 136848114 | 136853357 | CCATTTTA | CCATTTTA |
| CM-Zm510 | chr9 | 138588881 | 138607634 | CCTCTCCCA | CCTCTCCCA |
| CM-Zm511 | chr9 | 139196523 | 139213100 | TACAGGTAAG | TACAGGTAAG |
| CM-Zm512 | chr9 | 144625513 | 144630537 | CGAGGCACC | CGAGGCACC |
| CM-Zm513 | chr9 | 144924739 | 144953166 | CCGAGCACG | CCGAGCACG |
| CM-Zm514 | chr9 | 14654148 | 14663031 | GGACGGTGA | GGACGGTGA |
| CM-Zm515 | chr9 | 14785026 | 14796660 | GAACAGCTG | GAACAGCTG |
| CM-Zm516 | chr9 | 148233100 | 148236218 | GCTGGAGCG | GCTGGAGCG |
| CM-Zm517 | chr9 | 154251748 | 154280314 | CCGTCATA | CCGTCATA |
| CM-Zm518 | chr9 | 154370506 | 154373995 | GTCAGATCGG | ATCAGATCAG |
| CM-Zm519 | chr9 | 16469575 | 16473784 | TTATTTTTA | TTATTTTTA |
| CM-Zm520 | chr9 | 18696775 | 18702303 | ACTCGGGCT | CCTCGGACT |
| CM-Zm521 | chr9 | 20963686 | 20968599 | TCCAGAAAA | TCCAGAAAA |
| CM-Zm522 | chr9 | 23956291 | 23959193 | GCCTAGGTC | GCCTGGGTC |
| CM-Zm523 | chr9 | 36614213 | 36623365 | TCGCTATTC | TCGGTATTC |
| CM-Zm524 | chr9 | 4011555 | 4018753 | TATCTATTTA | TATCTATTTA |
| CM-Zm525 | chr9 | 4487196 | 4491733 | TGTTTAGAG | TGTTTAGAG |
| CM-Zm526 | chr9 | 61050572 | 61071849 | TATTTTCTA | TATTTTCTA |
| CM-Zm527 | chr9 | 65828484 | 65831964 | TTTAAATTA | TTTAAATTA |
| CM-Zm528 | chr9 | 712107 | 723868 | GAAACAAATG | GAAACAAATG |
| CM-Zm529 | chr9 | 81031876 | 81053042 | TGGGGACGG | TGGGGACGG |
| CM-Zm530 | chr9 | 82821014 | 82833419 | TTAAGTAA | TTAAGTAA |
| CM-Zm531 | chr9 | 87350125 | 87359215 | CCGCCGTCT | CCGCCGTCT |
| CM-Zm074 | chr10 | 102359621 | 102366284 | TACCAAGTA | TACCAAGTA |
| CM-Zm075 | chr10 | 10841629 | 10879341 | TAAATTTAA | TAAATTTAA |
| CM-Zm076 | chr10 | 119055932 | 119061702 | AGAGAAGG | AGAGAAGG |
| CM-Zm077 | chr10 | 122171426 | 122175923 | CCCGCAGGC | CCCGTCGGC |
| CM-Zm078 | chr10 | 12364522 | 12383225 | TTGAATAA | TTAAATAA |
| CM-Zm079 | chr10 | 124669166 | 124673487 | AAATAGAGG | AAATAGAGG |
| CM-Zm080 | chr10 | 125546015 | 125549913 | AAAAAAAAG | CAAAAAAAC |
| CM-Zm081 | chr10 | 127349922 | 127355809 | TTCCTGTCG | TTCCTGTCG |
| CM-Zm082 | chr10 | 131340180 | 131371495 | GTCTCCCGT | GTCCCGCCT |
| CM-Zm083 | chr10 | 131655019 | 131712229 | CTCCCGTGT | GTCCCGCGT |
| CM-Zm084 | chr10 | 132918272 | 132953844 | TCATCTGAG | TCATCTCAG |
| CM-Zm085 | chr10 | 133356916 | 133366139 | TTTGCTTCA | TTTGCTTCA |
| CM-Zm086 | chr10 | 134870682 | 134915852 | CGACCACGC | CGACCACGA |
| CM-Zm087 | chr10 | 135497966 | 135507087 | CAGAAAAAA | CAGAAAAAA |
| CM-Zm088 | chr10 | 137644251 | 137647043 | TGAAAGTAA | TGAAAGTAA |
| CM-Zm089 | chr10 | 137834167 | 137839525 | TGGACCTGT | TGGACCTGT |
| CM-Zm090 | chr10 | 139126216 | 139191569 | ACCCGTGTG | ACCCGTGTG |
| CM-Zm091 | chr10 | 140730620 | 140733158 | AAAAAAATA | AAAAAAATA |
| CM-Zm092 | chr10 | 141582689 | 141585764 | GTCAGCCAC | GTCAGCCAC |
| CM-Zm093 | chr10 | 142078637 | 142090980 | GGGGGCGAC | GGGGGCGAC |
| CM-Zm094 | chr10 | 143260924 | 143265288 | TTTTTATTA | TTTTTATTA |
| CM-Zm095 | chr10 | 143592761 | 143596040 | ATGTGCGGTG | ATGTGCGGTG |
| CM-Zm096 | chr10 | 145904139 | 145920888 | GCCGACCG | GCCGACCG |
| CM-Zm097 | chr10 | 148866490 | 148870122 | GCCTGGGTCG | GCCTGGGTCG |
| CM-Zm098 | chr10 | 15205050 | 15208559 | TCCACCAAG | TCCACCAAG |
| CM-Zm099 | chr10 | 1633491 | 1638087 | CTGGGTGGG | CTGGGTGGG |
| CM-Zm100 | chr10 | 18351355 | 18369935 | CACGGGCACA | CACGGACACA |
| CM-Zm101 | chr10 | 21864112 | 21870888 | GCAAAACAA | GCAAAACAA |
| CM-Zm102 | chr10 | 22419880 | 22426766 | GCGAGTATA | GCGGGTATA |
| CM-Zm103 | chr10 | 22624106 | 22632667 | CATCTTCTG | CATCTTCTG |
| CM-Zm104 | chr10 | 2436956 | 2446096 | TCTGCTCGG | TCTGATCGG |
| CM-Zm105 | chr10 | 4050403 | 4053261 | TAGGGAGGA | TAGGGAGGA |
| CM-Zm106 | chr10 | 42555380 | 42563704 | ACGTGAGGT | ACGTGAGGT |
| CM-Zm107 | chr10 | 4439571 | 4464012 | CTTTATTTA | CTTTATTTA |
| CM-Zm108 | chr10 | 46786147 | 46791890 | ATCAATGAG | ATCAATGGG |
| CM-Zm109 | chr10 | 57466453 | 57508226 | GCTGGGAAG | GCTGGGCAAG |
| CM-Zm110 | chr10 | 5877780 | 5880281 | TAATTTAAA | TAATTTAAA |
| CM-Zm111 | chr10 | 719135 | 729721 | TATATATTA | TATATATTA |
| CM-Zm112 | chr10 | 68129098 | 68141928 | TGGACAAGCTA | TGGACAAGCTA |
| CM-Zm113 | chr10 | 69433805 | 69459974 | ACCTACCG | ACCTACCG |
| CM-Zm114 | chr10 | 81898682 | 81956839 | GTGCATAGG | GTTCATAGG |
| CM-Zm115 | chr10 | 82236180 | 82239705 | GTTTGGTTG | GTTTGGTTG |
| CM-Zm116 | chr10 | 85301587 | 85309941 | ATAATTATA | ATAATTTTA |
| CM-Zm117 | chr10 | 85416582 | 85465252 | CGTACATC | CGTACATC |
| CM-Zm118 | chr10 | 90037589 | 90044599 | GACCAGCCGT | GAACAGCCGT |
| CM-Zm119 | chr10 | 95693961 | 95713045 | CGCACGTGGT | CGCACGTGGT |
| CM-Zm120 | chr10 | 98499894 | 98503076 | GTTATTCTA | GTTATTCTA |
| Table S2B. Genomic coordinates and target site duplications (TSDs) of the candidate coding-MULEs in rice. | | | | | |
| Coding-MULE | Chromosome | Start | End | TSD-left | TSD-right |
| CM-Os001 | chr1 | 10380647 | 10387428 | GCGAGGGTA | GCGAGGGTA |
| CM-Os002 | chr1 | 10388424 | 10396462 | AATTTTGTGA | AATTTTATGA |
| CM-Os003 | chr1 | 11561481 | 11569706 | TCTTTTACC | TCTTTTACC |
| CM-Os004 | chr1 | 11815917 | 11824465 | GTTTTATG | GTTTTATG |
| CM-Os005 | chr1 | 12249843 | 12257897 | AAATAAAT | AAATAAAT |
| CM-Os006 | chr1 | 12393542 | 12398628 | TGTGCATGC | TGTGCATGC |
| CM-Os007 | chr1 | 12776614 | 12804315 | TTTTTAAA | TTTTTAAA |
| CM-Os008 | chr1 | 13004483 | 13009931 | TTAAAGAT | TTAAAGAT |
| CM-Os009 | chr1 | 13154645 | 13169260 | TGTGGAAGT | TGTGGAAGT |
| CM-Os010 | chr1 | 14999791 | 15005292 | CCTTATTTA | CCTTATTTA |
| CM-Os011 | chr1 | 15196635 | 15205754 | GAGTTTACA | GAGTTTACA |
| CM-Os012 | chr1 | 15505241 | 15507820 | AATCTAACC | AATTGAACC |
| CM-Os013 | chr1 | 15768743 | 15777339 | CTATCCCCT | CTATCCCCT |
| CM-Os014 | chr1 | 15917560 | 15919939 | TAATAAAAA | TAATTAAAA |
| CM-Os015 | chr1 | 16089127 | 16095239 | CATAGAAAT | CATAGAAAT |
| CM-Os016 | chr1 | 16664539 | 16671903 | GTACGTACA | GTACGTACA |
| CM-Os017 | chr1 | 17416866 | 17419794 | AAAATTAG | AAAATTAG |
| CM-Os018 | chr1 | 17472118 | 17477749 | GGGGAGTGGG | GGGGAGTGGG |
| CM-Os019 | chr1 | 19031984 | 19036650 | AAAAAAAA | AAAAAAAA |
| CM-Os020 | chr1 | 19493909 | 19502775 | GAAGCCGC | GAAGCCGC |
| CM-Os021 | chr1 | 19895199 | 19899881 | TAACAAAA | TAACAAAA |
| CM-Os022 | chr1 | 19854809 | 19859812 | ACGGAGTG | ACGGAGTG |
| CM-Os023 | chr1 | 20173144 | 20177695 | TAATTTTC | TAATTTTC |
| CM-Os024 | chr1 | 20869374 | 20873152 | AGAGGAAGA | AGATGAAGA |
| CM-Os025 | chr1 | 22528258 | 22531107 | CAGCAGCA | CAGCAGCA |
| CM-Os026 | chr1 | 2298685 | 2306373 | GTCAGTGCT | GTCAGTGCT |
| CM-Os027 | chr1 | 22697290 | 22705235 | GAGGAGAGAA | GAGGGGAGAA |
| CM-Os028 | chr1 | 23124861 | 23143371 | GACATGGCA | GACATGGCA |
| CM-Os029 | chr1 | 24486442 | 24496062 | TTGTTTTC | TTGTTTTC |
| CM-Os030 | chr1 | 25196360 | 25203051 | GCCAGTCCG | GCCAGTCCG |
| CM-Os031 | chr1 | 25629294 | 25636742 | AATGAGGTTG | AATCAGGTTG |
| CM-Os032 | chr1 | 26567323 | 26571893 | ATCAGAGTT | ATCAGAGTT |
| CM-Os033 | chr1 | 26659223 | 26679353 | GTCGCAAAA | GTCGCAAAA |
| CM-Os034 | chr1 | 26835621 | 26843407 | TGCAGCCGT | TGCAGCCGT |
| CM-Os035 | chr1 | 27345029 | 27352986 | TCTGAATGT | TCTGAATGT |
| CM-Os036 | chr1 | 28224114 | 28231415 | CAGGGGTGT | CAGGGGTGT |
| CM-Os037 | chr1 | 28699044 | 28704650 | CACTCTGT | CACTCTGT |
| CM-Os038 | chr1 | 29178747 | 29182855 | CCACACGA | CCACACGA |
| CM-Os039 | chr1 | 29415346 | 29423397 | GTGCTCCGT | GTGCTCCGT |
| CM-Os040 | chr1 | 29369415 | 29377125 | CACTTTTGGC | CACTTTTGGC |
| CM-Os041 | chr1 | 2989452 | 2997976 | TGCTACTG | TGCTACTG |
| CM-Os042 | chr1 | 30640087 | 30644634 | TCTGGAAGC | TCTGGCAGC |
| CM-Os043 | chr1 | 31186503 | 31195565 | TTAGTATTAT | TTAGTACTAT |
| CM-Os044 | chr1 | 31671829 | 31676560 | GAGGTGTGT | GAGGTGGGT |
| CM-Os045 | chr1 | 3246256 | 3250270 | CTTTACAT | CTTTACAT |
| CM-Os046 | chr1 | 32392727 | 32418025 | ATGCCCTCAC | AGGCCCTCAA |
| CM-Os047 | chr1 | 32466640 | 32470222 | GAATTTGAA | GAATTTGAA |
| CM-Os048 | chr1 | 33442133 | 33447535 | CTGCGGGCT | CTGCGGGCT |
| CM-Os049 | chr1 | 34424794 | 34427758 | GGCGGTAGAT | GGCGGTAGAT |
| CM-Os050 | chr1 | 35208197 | 35216129 | CCGCGTTG | CCGCGTTG |
| CM-Os051 | chr1 | 35351964 | 35361116 | AAAAAATCT | AAAAAATCT |
| CM-Os052 | chr1 | 36312802 | 36321644 | TAATGATGGG | TAATCATGGG |
| CM-Os053 | chr1 | 36609927 | 36614940 | GTTGGCTT | GTTGGCTT |
| CM-Os054 | chr1 | 39511317 | 39515670 | GTCTACAGG | GTCTACAGG |
| CM-Os055 | chr1 | 39646798 | 39654516 | GAGGTAGGC | GAGGTAGGC |
| CM-Os056 | chr1 | 39765496 | 39772920 | GCCCGCCAG | GCCCGCCAG |
| CM-Os057 | chr1 | 41650106 | 41657907 | CACGTTTGT | CACGTTTGT |
| CM-Os058 | chr1 | 41832740 | 41835305 | GTTTCCAAC | GTTTCCAAC |
| CM-Os059 | chr1 | 42804036 | 42811839 | CTAAATAG | CTAAATAG |
| CM-Os060 | chr1 | 42829413 | 42837495 | GTACTTTTT | GTACTTTTT |
| CM-Os061 | chr1 | 4834979 | 4846219 | TGCTATACGT | TCCTATATGT |
| CM-Os062 | chr1 | 5176127 | 5183519 | TGATTTTGC | TGATTTTGC |
| CM-Os063 | chr1 | 6297826 | 6301637 | CTTAGAAA | CTTAGAAA |
| CM-Os064 | chr1 | 6545943 | 6555093 | TTACTACG | TTACTACG |
| CM-Os065 | chr1 | 8006104 | 8010164 | CAAGAGAA | CAAGAGAA |
| CM-Os066 | chr1 | 8774642 | 8781441 | CTATTGCT | CTATTGCT |
| CM-Os067 | chr1 | 8804287 | 8809357 | GCCATCTAT | GCCATCTAT |
| CM-Os068 | chr1 | 9029148 | 9035443 | TAATTGGGT | TAATTGGGT |
| CM-Os069 | chr1 | 9279170 | 9282622 | TTGCATCTTGG | TTGCATCTTGG |
| CM-Os168 | chr2 | 10100363 | 10106100 | TTTTTATAC | TTTTTATAC |
| CM-Os169 | chr2 | 10680634 | 10688958 | CAGTTGCTG | CAGTTGCTG |
| CM-Os170 | chr2 | 1295114 | 1299967 | TCAAAGTGTC | TCAAGTGTC |
| CM-Os171 | chr2 | 1351355 | 1358988 | AGAGACGA | AGAGACGA |
| CM-Os172 | chr2 | 13132692 | 13137436 | TTGAATTT | TTGAATTT |
| CM-Os173 | chr2 | 16155825 | 16161296 | TAAAAAAAAA | TAAAAAATAA |
| CM-Os174 | chr2 | 16281840 | 16287716 | GCTACTCGC | GCTACTCGC |
| CM-Os175 | chr2 | 16289385 | 16297392 | CTCCTTCCC | CTCCTTCCC |
| CM-Os176 | chr2 | 16431566 | 16440099 | GGCAATCGC | GGCAATCGC |
| CM-Os177 | chr2 | 17419770 | 17426340 | AGCTCTTAA | AGCTCTTAA |
| CM-Os178 | chr2 | 17613860 | 17631397 | AATTTTAAA | AATTTTAAA |
| CM-Os179 | chr2 | 1839938 | 1847558 | CGCGACAT | CGCGACAT |
| CM-Os180 | chr2 | 18146875 | 18167148 | TTGCATAAA | TTGCAGAAA |
| CM-Os181 | chr2 | 2166632 | 2174533 | TCCTCGCGG | TCCTCGCGG |
| CM-Os182 | chr2 | 21502499 | 21513914 | TTTATATA | TTTATATA |
| CM-Os183 | chr2 | 2265032 | 2274113 | TCTTTCTTA | TCTTTCTTA |
| CM-Os184 | chr2 | 2432595 | 2444312 | CTCAAAAAC | CTCAAAAAC |
| CM-Os185 | chr2 | 24108113 | 24114856 | CAAATTCG | CAAATTCG |
| CM-Os186 | chr2 | 25167080 | 25175121 | TTAGGCCAA | TTAGGCCAA |
| CM-Os187 | chr2 | 27675074 | 27685259 | GCGGAAGC | GCGGAAGC |
| CM-Os188 | chr2 | 27713925 | 27721727 | GCGGAAGC | GCGGAAGC |
| CM-Os189 | chr2 | 28146347 | 28151449 | TTATATTA | TTATATTA |
| CM-Os190 | chr2 | 28184816 | 28190969 | CCGCCACC | CCGCCACC |
| CM-Os191 | chr2 | 28456668 | 28463936 | CAAAAGCTGG | CAAATGCTGG |
| CM-Os192 | chr2 | 29002218 | 29007076 | GCGGGAGCAC | GAGGGAGCAC |
| CM-Os193 | chr2 | 2986899 | 2995453 | CACATTTGTA | CGCATTTGTA |
| CM-Os194 | chr2 | 29923443 | 29927496 | ATGAATCA | ATGAATCA |
| CM-Os195 | chr2 | 3043033 | 3048302 | CTCCACGG | CTCCACGG |
| CM-Os196 | chr2 | 354800 | 384612 | GTACTATGG | GTACTATGG |
| CM-Os197 | chr2 | 31853609 | 31862093 | ACCAAAAT | ACCAAAAT |
| CM-Os198 | chr2 | 33658494 | 33662653 | GTTATTAAA | GTTATTAAA |
| CM-Os199 | chr2 | 34521042 | 34529629 | GAAAATTCC | GAAAATTCC |
| CM-Os200 | chr2 | 35649458 | 35656637 | TGCAATAGG | TGCAATAGG |
| CM-Os201 | chr2 | 35730947 | 35738769 | CATATTCA | CATATTCA |
| CM-Os202 | chr2 | 476991 | 480465 | GTGCCCTTT | GTGCCGTTT |
| CM-Os203 | chr2 | 4670011 | 4676591 | AATTTCCA | AATTTCCA |
| CM-Os204 | chr2 | 5168269 | 5176304 | GATGGAGT | GATGGAGT |
| CM-Os205 | chr2 | 5789299 | 5797205 | GTGGAGTGC | GCGGAGTGC |
| CM-Os206 | chr2 | 5959533 | 5965411 | TAAATTTT | TAAATTTT |
| CM-Os207 | chr2 | 6040743 | 6049053 | GCTTTCGGA | GCTTTCGGA |
| CM-Os208 | chr2 | 8734016 | 8743087 | TTAAGAAA | TTAAGAAA |
| CM-Os209 | chr2 | 9122385 | 9130952 | ACAGAGATG | ACAGAGATG |
| CM-Os210 | chr2 | 955916 | 961469 | TCACTAAA | TCACTAAA |
| CM-Os211 | chr3 | 10064378 | 10071776 | CCTGCAGG | CCTGCAGG |
| CM-Os212 | chr3 | 10664573 | 10672388 | ATGAAACAAC | ATGAAACAAC |
| CM-Os213 | chr3 | 10738780 | 10745688 | CATGCGGCG | CATGCGGCG |
| CM-Os214 | chr3 | 11416805 | 11421472 | TATCATAAA | TATAATAAA |
| CM-Os215 | chr3 | 11830710 | 11835058 | TATTAAAT | TATTAAAT |
| CM-Os216 | chr3 | 1310008 | 1315232 | TAAAAAGAA | AAAGAAGAA |
| CM-Os217 | chr3 | 12362493 | 12367707 | GGGGATGG | GGGGATGG |
| CM-Os218 | chr3 | 12885182 | 12893702 | GAACAAAT | GAACAAAT |
| CM-Os219 | chr3 | 14026410 | 14029341 | GCATGCATG | GCATGCATG |
| CM-Os220 | chr3 | 16129988 | 16135176 | TTAAAAAAA | TTAAAAAAA |
| CM-Os221 | chr3 | 16228733 | 16237859 | CGGAGAGC | CGGAGAGC |
| CM-Os222 | chr3 | 17022860 | 17034328 | GATTCGTA | GATTCGTA |
| CM-Os223 | chr3 | 1898253 | 1902843 | GGTGTGGA | GGTGTGGA |
| CM-Os224 | chr3 | 18698255 | 18706858 | CTTTTCCCC | CTTTTCCCC |
| CM-Os225 | chr3 | 1962703 | 1967935 | TCCGGCGA | TCCGGCGA |
| CM-Os226 | chr3 | 19845839 | 19861934 | TAATAATAATA | TAATAATAATA |
| CM-Os227 | chr3 | 21554393 | 21562291 | CTATATGA | CTATATGA |
| CM-Os228 | chr3 | 22152565 | 22159589 | ATTTGATTT | ATTTGATTT |
| CM-Os229 | chr3 | 23118580 | 23123964 | TTAATATA | TTAATATA |
| CM-Os230 | chr3 | 25106734 | 25112001 | TTTTTGTT | TTTTTGTT |
| CM-Os231 | chr3 | 2630154 | 2637957 | GAAGATCC | GAAGATCC |
| CM-Os232 | chr3 | 26057410 | 26063090 | TATAGATGA | TATAGATGA |
| CM-Os233 | chr3 | 26370439 | 26377541 | CTTATTCCA | CTTATTCCA |
| CM-Os234 | chr3 | 26487140 | 26494943 | ACTTGTTA | ACTTGTTA |
| CM-Os235 | chr3 | 26668507 | 26675878 | TTGTAGCAG | TTGTAGCAG |
| CM-Os236 | chr3 | 27975349 | 27983739 | TCTCCTCG | TCTCCTCG |
| CM-Os237 | chr3 | 28199672 | 28216849 | CCATTGCAC | CCATTGCAC |
| CM-Os238 | chr3 | 2887953 | 2894357 | GTTGAATT | GTTGAATT |
| CM-Os239 | chr3 | 30230239 | 30235716 | CCAAAAGAT | CCAAAAGAT |
| CM-Os240 | chr3 | 30467549 | 30475447 | GAGTCGCGA | GAGTCGCGA |
| CM-Os241 | chr3 | 31699304 | 31708860 | TCACACAT | TCACACAT |
| CM-Os242 | chr3 | 34562591 | 34565520 | TTTTCGGTA | TTTTCGGTA |
| CM-Os243 | chr3 | 34863505 | 34867752 | TAAGGGTGAG | TAAGGGTGAG |
| CM-Os244 | chr3 | 35064892 | 35069303 | CAAAAGGA | CAAAAGGA |
| CM-Os245 | chr3 | 35224289 | 35232901 | ATCGTGCAA | ATCGTGCAA |
| CM-Os246 | chr3 | 35325902 | 35329239 | AAACACTGC | AAACACTGC |
| CM-Os247 | chr3 | 35527018 | 35537704 | CTAAATTT | CTAAATTT |
| CM-Os248 | chr3 | 35639579 | 35645215 | GTTGATACC | GTTGATACC |
| CM-Os249 | chr3 | 591953 | 598694 | GCAATTGTT | GCAATTGTT |
| CM-Os250 | chr3 | 6065613 | 6070396 | TAATAATAAT | TAATAATAAT |
| CM-Os251 | chr3 | 6187960 | 6194472 | GAGAGAGGA | GAGAGAGGA |
| CM-Os252 | chr3 | 7417254 | 7426405 | GGATTTTT | GGATTTTT |
| CM-Os253 | chr3 | 7457809 | 7465714 | GGATTTTT | GGATTTTT |
| CM-Os254 | chr3 | 818739 | 837241 | GTGTTGGAA | GTGTTGGAA |
| CM-Os255 | chr3 | 9105045 | 9124389 | GAGATTACG | AAGACTACG |
| CM-Os256 | chr3 | 9174657 | 9177608 | TCCGGATGA | TCCGGATGA |
| CM-Os257 | chr4 | 10150622 | 10158559 | CCCTAAGT | CCCTAAGT |
| CM-Os258 | chr4 | 13414620 | 13422112 | CCGGCGTTC | CCGGCGTTC |
| CM-Os259 | chr4 | 13904597 | 13909141 | CACTTGTTA | CACTTGTTA |
| CM-Os260 | chr4 | 14191737 | 14199316 | GTGTTTCC | GTGTTTCC |
| CM-Os261 | chr4 | 14496157 | 14514315 | TCCAAAAA | TCCAAAAA |
| CM-Os262 | chr4 | 14622595 | 14630624 | TTAGAGAA | TTAGAGAA |
| CM-Os263 | chr4 | 14653601 | 14658105 | CAAACCTAT | CAAACCTAT |
| CM-Os264 | chr4 | 17592266 | 17603906 | CAATATTTGG | CAATATTTGG |
| CM-Os265 | chr4 | 17848532 | 17865097 | GTAAGTAGG | GTAAGTAGG |
| CM-Os266 | chr4 | 18016831 | 18023574 | CAAACAACG | CAAACAACG |
| CM-Os267 | chr4 | 18509718 | 18517638 | ACATGAGATG | ACATGAGATG |
| CM-Os268 | chr4 | 18959501 | 18964760 | CAAGAGCAA | CAAGAGCAA |
| CM-Os269 | chr4 | 22752934 | 22759703 | CTATCTATCTA | CTATCTATCTA |
| CM-Os270 | chr4 | 23086426 | 23092371 | GAGCTTTTT | GAGCTTTTT |
| CM-Os271 | chr4 | 23990171 | 23998705 | TTCGGACCG | TTCGGACCG |
| CM-Os272 | chr4 | 2449002 | 2452025 | ATGTAGAC | ATGTAGAC |
| CM-Os273 | chr4 | 24454403 | 24459484 | TAGGATAT | TAGGATAT |
| CM-Os274 | chr4 | 24712357 | 24717017 | CTCCACTGC | CTCCACTGC |
| CM-Os275 | chr4 | 24892206 | 24894300 | TCCGTCTCTT | TCCGTCTCTT |
| CM-Os276 | chr4 | 25735533 | 25744111 | TTGGGGGAG | TTGGGGGAG |
| CM-Os277 | chr4 | 25963641 | 25970702 | ACCAGATGA | ACCAGATGA |
| CM-Os278 | chr4 | 2746631 | 2749146 | CCCTCTCT | CCCTCTAT |
| CM-Os279 | chr4 | 27489281 | 27492504 | CCGCCGGC | CCGCCGGC |
| CM-Os280 | chr4 | 28358156 | 28366061 | GATCCGCC | GATCCGCC |
| CM-Os281 | chr4 | 29198704 | 29204181 | TATCTAAA | TATCTAAA |
| CM-Os282 | chr4 | 30486272 | 30495212 | TCCCGTCTCC | TCCCGTCTCC |
| CM-Os283 | chr4 | 31568528 | 31577115 | CGGCCGCGT | CGGCCGCGT |
| CM-Os284 | chr4 | 31616554 | 31629052 | TTCGGTTTGC | TTCGGTTTGC |
| CM-Os285 | chr4 | 31842272 | 31847773 | GCTAAACGC | GCTAAACGC |
| CM-Os286 | chr4 | 32067389 | 32075184 | GGAAAAAGT | GGAAAAAGT |
| CM-Os287 | chr4 | 32213972 | 32221745 | GATGAGCCGT | GATGAGCCGT |
| CM-Os288 | chr4 | 32559063 | 32564348 | CTGGATTGT | CTGGATTGT |
| CM-Os289 | chr4 | 32979624 | 32987158 | TAGCTTTGG | TAGCTTTGG |
| CM-Os290 | chr4 | 33086864 | 33090077 | GATGAATCT | GATGAATCT |
| CM-Os291 | chr4 | 33613531 | 33621585 | CCGCTGCAC | CCGCTGCAC |
| CM-Os292 | chr4 | 34631354 | 34639214 | GCCTTCCAT | GCCTTCCAT |
| CM-Os293 | chr4 | 35302323 | 35310223 | GTCTTTTCT | GTCTTTTCT |
| CM-Os294 | chr4 | 4135346 | 4142609 | ATCGTTCAG | ATCGTTCAG |
| CM-Os295 | chr4 | 5365498 | 5373311 | CTCCTTTG | CTCCTTTG |
| CM-Os296 | chr4 | 5631117 | 5635544 | ACCAGCGGG | ACCAGGGGG |
| CM-Os297 | chr4 | 827326 | 835325 | CTCGTTCGG | CTCGTTCGG |
| CM-Os298 | chr4 | 848571 | 856255 | GTACGTACG | ATACGTACG |
| CM-Os299 | chr4 | 9720294 | 9740041 | CCCGATTGA | CCCGATTGA |
| CM-Os300 | chr4 | 10027954 | 10047108 | TGAGAAGCG | CGGGAAGCG |
| CM-Os301 | chr5 | 11002429 | 11005319 | CCCCCCCC | CCCCCCCC |
| CM-Os302 | chr5 | 14520200 | 14525602 | TCCCCCAAA | TCCACCAAA |
| CM-Os303 | chr5 | 15007690 | 15021232 | CTATGTAAAC | CTATGTAAAC |
| CM-Os304 | chr5 | 15112821 | 15124583 | GTATGCGCA | GTATGCGCA |
| CM-Os305 | chr5 | 15188785 | 15193266 | ATTAAAAA | ATTAAAAA |
| CM-Os306 | chr5 | 16445971 | 16454496 | ACGAGTGTT | ACGAGTGTT |
| CM-Os307 | chr5 | 1688925 | 1696567 | AAAATCTA | AAAATCTA |
| CM-Os308 | chr5 | 17483703 | 17488485 | GAGATCGGA | GAGATCGGA |
| CM-Os309 | chr5 | 17573195 | 17577179 | TTCCATCT | TTCCATCT |
| CM-Os310 | chr5 | 18419277 | 18425016 | AAAAGGGG | AAAAGGGG |
| CM-Os311 | chr5 | 18973617 | 18978437 | CTCAGGTG | CTCAGGTG |
| CM-Os312 | chr5 | 19594838 | 19606550 | TCGTCAAAT | TCGTCAAAT |
| CM-Os313 | chr5 | 2067211 | 2071542 | GGCGTATGA | GGCGTATGA |
| CM-Os314 | chr5 | 21157739 | 21160836 | CGCCTCTACC | CGCTTCTTCC |
| CM-Os315 | chr5 | 21218467 | 21222139 | CTTACTCGC | CTTACTCGC |
| CM-Os316 | chr5 | 21354481 | 21361774 | GCTTGCTTG | GCTTGCTTG |
| CM-Os317 | chr5 | 22178735 | 22181892 | GTTTTTTTA | GTTTTTTTA |
| CM-Os318 | chr5 | 22415635 | 22423439 | GTCGCCGCT | GTCGCCGCT |
| CM-Os319 | chr5 | 23373898 | 23377943 | CCAGCGAG | CCAGCGAG |
| CM-Os320 | chr5 | 23813086 | 23816764 | CTTCTGGAA | CTTCTGGAA |
| CM-Os321 | chr5 | 23889866 | 23897347 | CGCGTGTTG | CGCGTGTTG |
| CM-Os322 | chr5 | 24050507 | 24055773 | TATTTTGA | TATTTTGA |
| CM-Os323 | chr5 | 2472984 | 2477805 | CATATAAATT | CATATAAATT |
| CM-Os324 | chr5 | 24330435 | 24342057 | GGCGGTGGAA | GGCTGTGGAA |
| CM-Os325 | chr5 | 24599984 | 24605292 | CCAGGACG | CCAGGACG |
| CM-Os326 | chr5 | 25045736 | 25052480 | CCCCATCGG | CCCCATCGG |
| CM-Os327 | chr5 | 25169791 | 25174723 | TTTTTTTAA | TTTTTTTTCA |
| CM-Os328 | chr5 | 25234956 | 25252277 | AAACTGGTC | AAACTGGTC |
| CM-Os329 | chr5 | 25997387 | 26014903 | TTATTTTAG | TTATTTTAG |
| CM-Os330 | chr5 | 26668736 | 26675209 | CCCAAACAA | CCCAAACAA |
| CM-Os331 | chr5 | 26999234 | 27020326 | ATTCAAAC | ATTCAAAC |
| CM-Os332 | chr5 | 27331635 | 27343595 | TGCAACTT | TGCAACTT |
| CM-Os333 | chr5 | 29083103 | 29092346 | CCGTCATCCC | CCGTCATCCC |
| CM-Os334 | chr5 | 2999181 | 3003378 | CTACATCG | CTACATCG |
| CM-Os335 | chr5 | 464871 | 469841 | GTTTCAAG | GTTTCAAG |
| CM-Os336 | chr5 | 498630 | 505448 | AATAATAATA | AATAATAATA |
| CM-Os337 | chr5 | 4396037 | 4401841 | TCTCCTCT | TCTCCTCT |
| CM-Os338 | chr5 | 4636334 | 4644028 | GGCAAGAGA | GGCAAGAGA |
| CM-Os339 | chr5 | 9359638 | 9365958 | AATCTTGA | AATCTTGA |
| CM-Os340 | chr6 | 10495669 | 10548631 | TTTTTTTA | TTTTTTTA |
| CM-Os341 | chr6 | 10937072 | 10955192 | TCAATTTGG | TCAATTTGG |
| CM-Os342 | chr6 | 11422660 | 11426077 | CTCCCCTT | CTCCCCTT |
| CM-Os343 | chr6 | 11593307 | 11602460 | GAATTTATA | GAATTTATA |
| CM-Os344 | chr6 | 11951774 | 11956950 | TTTAAAAT | TTTAAAAT |
| CM-Os345 | chr6 | 12462962 | 12471473 | GACAACATG | GACAACATG |
| CM-Os346 | chr6 | 13206026 | 13214386 | CCCAAGAAC | CCCAAGAAC |
| CM-Os347 | chr6 | 13420225 | 13423303 | TTGACATGA | TTGACATGA |
| CM-Os348 | chr6 | 13557632 | 13565944 | GGACTCCTG | GGACTCCTG |
| CM-Os349 | chr6 | 14959056 | 14966833 | TATATAGGA | TATATAGGA |
| CM-Os350 | chr6 | 16520199 | 16524875 | ATTTGATT | ATTTGATT |
| CM-Os351 | chr6 | 17806835 | 17811851 | TTAATAAAA | TTGATAAAA |
| CM-Os352 | chr6 | 19230651 | 19235035 | ACTTTTCG | ACTTTTCG |
| CM-Os353 | chr6 | 2076675 | 2084479 | CATATCCCC | CATATCCCC |
| CM-Os354 | chr6 | 20891280 | 20895691 | GCAAGAGC | GCAAGAGC |
| CM-Os355 | chr6 | 21403428 | 21411211 | GTTGGGGGT | GTTGGGGGT |
| CM-Os356 | chr6 | 21846166 | 21854108 | GGTAGATGG | GGTAGATGG |
| CM-Os357 | chr6 | 22432756 | 22463437 | TTGGACTGG | TTGGACTGG |
| CM-Os358 | chr6 | 22730111 | 22733545 | TTCGTTTAG | TTCGTTTAG |
| CM-Os359 | chr6 | 23206290 | 23218096 | TATCATATT | TATCATATT |
| CM-Os360 | chr6 | 25133070 | 25142216 | GTAGATTAG | GTAGATTAG |
| CM-Os361 | chr6 | 25220708 | 25226344 | TTCCAATG | TTCCAATG |
| CM-Os362 | chr6 | 25479345 | 25487939 | TTAGAGGTA | TTAGAGGTA |
| CM-Os363 | chr6 | 26861512 | 26870665 | GAAAAGGGA | GAAAAGGGA |
| CM-Os364 | chr6 | 2817310 | 2826339 | ACGGGGAGA | ACGGGGAGA |
| CM-Os365 | chr6 | 29012298 | 29020241 | CAGCAAGA | CAGCAAGA |
| CM-Os366 | chr6 | 29999451 | 30008055 | TTGAATTGA | TTGAATTGA |
| CM-Os367 | chr6 | 30456373 | 30464151 | CTCGTCAA | CTCGTCAA |
| CM-Os368 | chr6 | 3903454 | 3910804 | CGCGCTACG | CGCGCTACG |
| CM-Os369 | chr6 | 4874900 | 4878759 | CGACACTCT | CGACACTCT |
| CM-Os370 | chr6 | 6046291 | 6050990 | GGAGAAGA | GGAGAAGA |
| CM-Os371 | chr6 | 6131230 | 6139415 | GTTTTGTGG | GTTTTGTGG |
| CM-Os372 | chr6 | 7061072 | 7066915 | TGCAAATAAA | TGCAAATAAA |
| CM-Os373 | chr6 | 7544380 | 7550010 | TTTAGCTA | TTTAGCTA |
| CM-Os374 | chr6 | 7971247 | 7978840 | CACCGGTTC | CACCGGTTC |
| CM-Os375 | chr6 | 8434195 | 8447856 | CGCGCTGG | CGCGCTGG |
| CM-Os376 | chr6 | 9180578 | 9185342 | TATTTTATA | TATTTTGTA |
| CM-Os377 | chr6 | 9413332 | 9415413 | GGGGGGTGTG | GGGGGGTGTG |
| CM-Os378 | chr7 | 10161227 | 10169445 | GTTCTCTCT | GTTCTCTCT |
| CM-Os379 | chr7 | 10683125 | 10690930 | CTCGAATAT | CTCGAATAT |
| CM-Os380 | chr7 | 11669997 | 11674349 | CGCTCCTG | CGCTCCTG |
| CM-Os381 | chr7 | 11792496 | 11804302 | CTCGCACTC | CTCGCACTC |
| CM-Os382 | chr7 | 1328672 | 1333825 | AAAAAACT | AAAAAACT |
| CM-Os383 | chr7 | 13058270 | 13064414 | ATGACACAAA | ATGACTCAAA |
| CM-Os384 | chr7 | 14998552 | 15006354 | TTCGTGGCA | TTCGTGGCA |
| CM-Os385 | chr7 | 16418914 | 16426948 | CACATCACG | CACATCACG |
| CM-Os386 | chr7 | 17430407 | 17434934 | ACTATGTAG | ACTATGTAG |
| CM-Os387 | chr7 | 17544330 | 17551431 | ATTATTTTG | ATTATTTTG |
| CM-Os388 | chr7 | 17856271 | 17865433 | GTGGTGTGA | GTGGTGTGA |
| CM-Os389 | chr7 | 17976478 | 17981685 | AATAAAGA | AATAAAGA |
| CM-Os390 | chr7 | 18035599 | 18040806 | AATAAAGA | AATAAAGA |
| CM-Os391 | chr7 | 1931302 | 1936337 | GGTGCTACC | GGTGCTACC |
| CM-Os392 | chr7 | 1975919 | 1985357 | AATCTAAAA | AATCTTAAA |
| CM-Os393 | chr7 | 19418690 | 19427865 | TATATTCT | TATATTCT |
| CM-Os394 | chr7 | 19673773 | 19681431 | AAGTGTTCA | AAGTGTTCA |
| CM-Os395 | chr7 | 20135462 | 20142941 | TCTCACTAT | TCTCACTAT |
| CM-Os396 | chr7 | 20550764 | 20559917 | TCCCTCCC | TCCCTCCC |
| CM-Os397 | chr7 | 2118384 | 2122966 | TCGCCTGCA | CCGCCTGCA |
| CM-Os398 | chr7 | 20991334 | 20999293 | CCTTCCAAG | CCTTCCAAG |
| CM-Os399 | chr7 | 22130964 | 22135197 | CCAAAGGAT | CCAAAGGAT |
| CM-Os400 | chr7 | 2267791 | 2274820 | GTCGGGTCGC | GTCGGGTCGC |
| CM-Os401 | chr7 | 22520335 | 22523196 | AAACATCAC | AAACATCAC |
| CM-Os402 | chr7 | 23632470 | 23636197 | AAAAAAGTT | AAAAAAGTT |
| CM-Os403 | chr7 | 23718512 | 23720914 | CTCCCCCACT | CTCCCCCCACT |
| CM-Os404 | chr7 | 24361650 | 24369453 | GTGTTTGGA | GTGTTTGGA |
| CM-Os405 | chr7 | 24425004 | 24429062 | TTGCTGAA | TTGCTGAA |
| CM-Os406 | chr7 | 24816157 | 24823431 | CCGCCTCTA | CCGCCTCTA |
| CM-Os407 | chr7 | 25264073 | 25271889 | GAAAACCAC | GAAAACCAC |
| CM-Os408 | chr7 | 25448537 | 25456131 | GAGAGATAG | GAGAGATAG |
| CM-Os409 | chr7 | 25746059 | 25752063 | GCCTTTTTAC | GCCTTTTTAC |
| CM-Os410 | chr7 | 26438609 | 26445392 | CACGGCGAC | CACGGCGAC |
| CM-Os411 | chr7 | 2772949 | 2781952 | CCGGGCGAA | CCTGGCGAA |
| CM-Os412 | chr7 | 27457665 | 27463160 | GTTCGATGC | GTTCGATGC |
| CM-Os413 | chr7 | 28088716 | 28096072 | CTTTAATT | CTTTAATT |
| CM-Os414 | chr7 | 4616461 | 4618566 | GATCTGTGT | GATCTGTGT |
| CM-Os415 | chr7 | 5630560 | 5635615 | GATGAATTT | GATGAATTT |
| CM-Os416 | chr7 | 7130558 | 7138269 | AATACAATC | AATACAATC |
| CM-Os417 | chr7 | 7371114 | 7379072 | CGTTTCGCT | CGTTTCGCT |
| CM-Os418 | chr7 | 7427161 | 7436861 | CTAGACAAA | CTAAACAAA |
| CM-Os419 | chr7 | 7674342 | 7683473 | CATGATCCCC | CATGATCCCC |
| CM-Os420 | chr8 | 10067147 | 10070614 | CCCCAAGCA | CCCCAAGCA |
| CM-Os421 | chr8 | 10438921 | 10443844 | TTTTTTTT | TTTTTTTT |
| CM-Os422 | chr8 | 15335661 | 15347882 | TAGATGAT | TAGATGAT |
| CM-Os423 | chr8 | 15560204 | 15566980 | ATGTGTTG | ATGTGTTT |
| CM-Os424 | chr8 | 15789724 | 15794865 | TAAAAAAA | TAAAAAAA |
| CM-Os425 | chr8 | 16102825 | 16110576 | TCTTTCCTT | TCTTTCCTT |
| CM-Os426 | chr8 | 16493311 | 16500599 | ATAGACCCA | ATAGACCCA |
| CM-Os427 | chr8 | 1733521 | 1742675 | TCTTTCAGGA | TCTTTCAGGA |
| CM-Os428 | chr8 | 17737691 | 17744960 | ACCTGGAG | ACCTGGAG |
| CM-Os429 | chr8 | 18462312 | 18467497 | CCGGACCGG | CCGGACCGG |
| CM-Os430 | chr8 | 19276769 | 19281872 | CTTAAAATA | CTCAAAATA |
| CM-Os431 | chr8 | 20105704 | 20110382 | GTAAAACG | GTAAAACG |
| CM-Os432 | chr8 | 20864015 | 20873454 | GAGGCGTCAC | GGGGCGTCAC |
| CM-Os433 | chr8 | 20948756 | 20966956 | GATTTGTA | GATTTGTA |
| CM-Os434 | chr8 | 21888542 | 21896349 | ATAGAGGAA | ATAGAGGAA |
| CM-Os435 | chr8 | 22679337 | 22684823 | GTGGGGTG | GTGGGGTG |
| CM-Os436 | chr8 | 23136940 | 23145893 | CGCGTCTC | CGCGTCTC |
| CM-Os437 | chr8 | 23160375 | 23164362 | TGCGCGCAA | TGCGCGCAA |
| CM-Os438 | chr8 | 2427309 | 2431345 | CTTGGAGGA | CTTAGAGAA |
| CM-Os439 | chr8 | 24101318 | 24108874 | TCGAAGCTG | TTGAAGCTG |
| CM-Os440 | chr8 | 25069954 | 25077348 | CTTCTCTCAA | CTTCTCCCAA |
| CM-Os441 | chr8 | 25987438 | 25996594 | GCGTGATCG | GCGTGATCG |
| CM-Os442 | chr8 | 26611329 | 26619869 | TTCAGTTGA | TTCAGTTGA |
| CM-Os443 | chr8 | 27078787 | 27085255 | TCGAATAG | TCGAATAG |
| CM-Os444 | chr8 | 2894207 | 2903358 | GGCTGATTT | GGCTGATTT |
| CM-Os445 | chr8 | 3805116 | 3814276 | GCTAACTCA | GCTAACTCA |
| CM-Os446 | chr8 | 5519463 | 5523620 | CTTCTGAG | CTTCTGAG |
| CM-Os447 | chr8 | 6275692 | 6279791 | TTGATTCTG | TTGATTCTG |
| CM-Os448 | chr8 | 7348222 | 7356851 | GCCGACACG | GCCGACACG |
| CM-Os449 | chr8 | 7856617 | 7858900 | AAGAGATGG | AAGAGATGG |
| CM-Os450 | chr8 | 8065514 | 8070395 | TAATTATT | TAATTATT |
| CM-Os451 | chr9 | 10054124 | 10059632 | TTTTTGAAA | TTTTTAAAT |
| CM-Os452 | chr9 | 10138554 | 10147123 | TACAGCTAT | TACAGCTAT |
| CM-Os453 | chr9 | 10198933 | 10202057 | GTCATATGT | GTCATATGT |
| CM-Os454 | chr9 | 11885989 | 11895816 | CCAAAACCA | CCAAAACCA |
| CM-Os455 | chr9 | 12235991 | 12244225 | TAATTGTA | TAATTGTA |
| CM-Os456 | chr9 | 13132439 | 13139758 | CGTGCGTTC | CGTGCGTTC |
| CM-Os457 | chr9 | 14067722 | 14071875 | ATCATTCGT | ATCATTCGT |
| CM-Os458 | chr9 | 1509296 | 1514669 | AAAGGAAATA | AAAGGAAATA |
| CM-Os459 | chr9 | 1520855 | 1539115 | TCATGATAAT | TGATGATAAT |
| CM-Os460 | chr9 | 15513123 | 15521109 | CTGCTTTGA | CTGCTTTGA |
| CM-Os461 | chr9 | 1659026 | 1668903 | TTCTTTCTC | TTCTTTCTC |
| CM-Os462 | chr9 | 16210132 | 16214620 | GTTTTCAA | GTTTTCAA |
| CM-Os463 | chr9 | 17197892 | 17203659 | CGCAGTTTA | CGCAGTTTA |
| CM-Os464 | chr9 | 18428055 | 18435503 | TAAAAAAA | TAAAAAAA |
| CM-Os465 | chr9 | 18477873 | 18485676 | GACCGACAA | GACCGACAA |
| CM-Os466 | chr9 | 19340607 | 19348412 | CTCAAGAAA | CTCAAGAAA |
| CM-Os467 | chr9 | 20575713 | 20579602 | TTGATTGG | TTGATTGG |
| CM-Os468 | chr9 | 21689475 | 21694569 | TCAATCTGC | TCAATCTGC |
| CM-Os469 | chr9 | 21784871 | 21791668 | TCCAATAAT | TCCAATAAT |
| CM-Os470 | chr9 | 22333545 | 22341482 | CCTTCTCT | CCTTCTCT |
| CM-Os471 | chr9 | 4477740 | 4483252 | TTAAATAT | TTAAATAT |
| CM-Os472 | chr9 | 5434361 | 5442955 | GTCATTTTT | GTCATTTTT |
| CM-Os473 | chr9 | 6167091 | 6174852 | CATTTTTT | CATTTTTT |
| CM-Os474 | chr9 | 665720 | 669714 | TCCTCTCT | TCCTCTCT |
| CM-Os475 | chr9 | 6596394 | 6598569 | TATTTTTA | TATTTTTA |
| CM-Os476 | chr9 | 7107981 | 7115919 | GTGTCCAGT | GTGTCCAGT |
| CM-Os070 | chr10 | 11452321 | 11460189 | AATGAACTT | AATGAACTT |
| CM-Os071 | chr10 | 12745023 | 12753005 | GGAAAAGC | GGAAAAGC |
| CM-Os072 | chr10 | 13665722 | 13673008 | GCTTGGTCA | GCTTGGTCA |
| CM-Os073 | chr10 | 14284407 | 14287731 | TTGCTCGT | TTGCCTCGT |
| CM-Os074 | chr10 | 14365299 | 14370078 | GTAAAGAAGG | GGATAGAAGG |
| CM-Os075 | chr10 | 14930259 | 14938155 | GCGATGATTCG | GCGATGATTCG |
| CM-Os076 | chr10 | 15702600 | 15707627 | TTTTATCA | TTTTATCA |
| CM-Os077 | chr10 | 16007045 | 16014836 | CATGTTCCA | CATGTTCCA |
| CM-Os078 | chr10 | 16444525 | 16450641 | TATAAATA | TATAAATA |
| CM-Os079 | chr10 | 17055260 | 17062974 | GCCTCCGGA | GCCTCCGGA |
| CM-Os080 | chr10 | 17422059 | 17431094 | GAGTTCCT | GAGTTCCT |
| CM-Os081 | chr10 | 18252934 | 18257968 | GTTCCGGT | GTTCCGGT |
| CM-Os082 | chr10 | 18892864 | 18896384 | CTCAATTTGC | CTCAATTTGC |
| CM-Os083 | chr10 | 19224126 | 19230297 | TAAAATGTT | TAAAATGTT |
| CM-Os084 | chr10 | 19440733 | 19448605 | GTAGGACTA | GTAGGACTA |
| CM-Os085 | chr10 | 20103926 | 20107874 | GTGCGCCTC | GTGCGCCTC |
| CM-Os086 | chr10 | 20639272 | 20645278 | TTGGTTTT | TTGGTTTT |
| CM-Os087 | chr10 | 22672707 | 22680057 | TTGTCTCT | TTGTCTCT |
| CM-Os088 | chr10 | 22956254 | 22964053 | CTGAATAT | CTGAATAT |
| CM-Os089 | chr10 | 23054943 | 23060706 | TTGATGCT | TTGATCCT |
| CM-Os090 | chr10 | 2934198 | 2939392 | TACAACTT | TACAACTT |
| CM-Os091 | chr10 | 3816010 | 3823293 | GGTCAACG | GGTCAACG |
| CM-Os092 | chr10 | 4038099 | 4043197 | TAGGGCTAT | TAGGGCTAT |
| CM-Os093 | chr10 | 5360308 | 5365251 | ATTTTCCCGC | ATTTACCCGC |
| CM-Os094 | chr10 | 7463320 | 7484514 | GGTAATTTGC | GATAATTTGC |
| CM-Os095 | chr10 | 7953756 | 7956117 | ATTCCAATT | ATTCCGATT |
| CM-Os096 | chr10 | 8948239 | 8953120 | TTTGTTTT | TTTGTTTT |
| CM-Os097 | chr10 | 944099 | 951966 | GAAACAGGAC | GAAACGGGAC |
| CM-Os098 | chr10 | 9204393 | 9212999 | AAAGGAACA | AAAGGAACA |
| CM-Os099 | chr10 | 9218513 | 9226447 | GTCTCCTTT | GTCTCCTTT |
| CM-Os100 | chr10 | 1039717 | 1047857 | CTTGCATCT | CTTGCATCT |
| CM-Os101 | chr11 | 1209766 | 1215293 | TATTTTAG | TATTTTAG |
| CM-Os102 | chr11 | 12732342 | 12743596 | CTGGGAGA | CTGGGAGA |
| CM-Os103 | chr11 | 14137161 | 14145765 | CAAAAGCAT | CAAAAGCAT |
| CM-Os104 | chr11 | 14535047 | 14543003 | CCCCGCCCCCC | CCCCGCCCCCC |
| CM-Os105 | chr11 | 15329360 | 15334158 | CATTATGC | CATTATGC |
| CM-Os106 | chr11 | 1598703 | 1601432 | GCGTCTCG | GCGTCTCG |
| CM-Os107 | chr11 | 17578513 | 17583438 | TTATAGGA | TTATAGGA |
| CM-Os108 | chr11 | 17536155 | 17544630 | TTCCCTCCC | TTCCCTCCC |
| CM-Os109 | chr11 | 17659827 | 17665081 | TTCACTCAA | TTCACTCAA |
| CM-Os110 | chr11 | 18627578 | 18635268 | TAACAATT | TAACAATT |
| CM-Os111 | chr11 | 18704626 | 18709292 | ATCTCGCGT | ATCTCGCGT |
| CM-Os112 | chr11 | 19137553 | 19145216 | CTTCTACTA | CTTCTACTA |
| CM-Os113 | chr11 | 19287924 | 19293333 | TTATTTTA | TTATTTTA |
| CM-Os114 | chr11 | 19404784 | 19412217 | TAGAATTTT | TAGAATTTT |
| CM-Os115 | chr11 | 19563327 | 19572472 | CTATCCAGC | CTATCAAGC |
| CM-Os116 | chr11 | 19686599 | 19693086 | CTCTATCGG | CTCTATCGG |
| CM-Os117 | chr11 | 21069912 | 21079063 | CGCGAATGC | CGCGAATGC |
| CM-Os118 | chr11 | 23363024 | 23372705 | TGAAATTCT | TGAAATTCT |
| CM-Os119 | chr11 | 24249538 | 24256341 | TAACAATT | TAACAATT |
| CM-Os120 | chr11 | 24975696 | 24980697 | TTTTAGTT | TTTTAGTT |
| CM-Os121 | chr11 | 26161816 | 26168555 | GATTGGTGG | GATTGGTGG |
| CM-Os122 | chr11 | 26349921 | 26356617 | CATATATTG | CATATATTG |
| CM-Os123 | chr11 | 26698195 | 26706131 | GTGATTCGC | GTGATTCGC |
| CM-Os124 | chr11 | 26864777 | 26872546 | CCACTAGAA | CCACGAAAA |
| CM-Os125 | chr11 | 28014577 | 28029697 | CTCAGCCCG | CTCAGCCCG |
| CM-Os126 | chr11 | 2988231 | 3017669 | GAGGGGAA | GAGGGGAA |
| CM-Os127 | chr11 | 3066147 | 3073623 | GTTCGGCGA | GTTCGGCGA |
| CM-Os128 | chr11 | 3526354 | 3530697 | AAATATTA | AAATATTA |
| CM-Os129 | chr11 | 4854839 | 4858973 | CTGGAAGGA | CTGGAAGGA |
| CM-Os130 | chr11 | 6207196 | 6211214 | GAGAAGAG | GAGAAGAG |
| CM-Os131 | chr11 | 7132555 | 7143218 | CTACAACCT | CTACAACCT |
| CM-Os132 | chr11 | 8143905 | 8152512 | GTCACCATG | GTCACCATG |
| CM-Os133 | chr11 | 8199126 | 8221838 | TAAAAGTAA | TAAAATAA |
| CM-Os134 | chr11 | 9942994 | 9947925 | AACAGAGA | AACAGAGA |
| CM-Os135 | chr12 | 10884052 | 10888903 | TAATTAAA | TAATTAAA |
| CM-Os136 | chr12 | 11305866 | 11314469 | GAAAAGTTG | GAAAAGTTG |
| CM-Os137 | chr12 | 12474380 | 12482266 | GCATCTTC | GCATCTTC |
| CM-Os138 | chr12 | 14037558 | 14045451 | TTAAGTAACA | TTAAGTAACA |
| CM-Os139 | chr12 | 1468705 | 1471956 | CAAATATCTA | CAAATATATA |
| CM-Os140 | chr12 | 15264610 | 15282869 | ATCAGGCG | ATCAGGCG |
| CM-Os141 | chr12 | 16607695 | 16613207 | ATGGGTTG | ATGGGTTG |
| CM-Os142 | chr12 | 16631664 | 16637176 | ATGGGTTG | ATGGGTTG |
| CM-Os143 | chr12 | 17588794 | 17596062 | CATGAAATT | CATGAAATT |
| CM-Os144 | chr12 | 17816780 | 17821497 | TTATTATT | TTATTATT |
| CM-Os145 | chr12 | 17993650 | 18002138 | GACAAAGA | GACAAAGA |
| CM-Os146 | chr12 | 19081331 | 19086880 | GCCAGACGT | GCCAGACGT |
| CM-Os147 | chr12 | 19095774 | 19100189 | TCTCAATTA | TCTCAATTA |
| CM-Os148 | chr12 | 19243252 | 19248965 | CTTCTCTCA | CTTCTCTCA |
| CM-Os149 | chr12 | 1977446 | 1983844 | GTAGACTGC | GTAGACTGC |
| CM-Os150 | chr12 | 19380970 | 19388596 | ATGTAATT | ATGTAATT |
| CM-Os151 | chr12 | 2100003 | 2118024 | GTGCTACAC | GTGCTACAC |
| CM-Os152 | chr12 | 21542591 | 21547000 | TTTCCACA | TTTCCACA |
| CM-Os153 | chr12 | 23643547 | 23655576 | TTCCTGTTC | TTCCTGTTC |
| CM-Os154 | chr12 | 23970111 | 23972322 | TTGATTTAA | TTGATTTAA |
| CM-Os155 | chr12 | 24073145 | 24083421 | GTGAAAAGGG | GTGAAAAGGG |
| CM-Os156 | chr12 | 24402018 | 24408489 | CAGAAGCG | CAGAAGCG |
| CM-Os157 | chr12 | 2628957 | 2633977 | GCCTTGAT | GCCTTGAT |
| CM-Os158 | chr12 | 25942583 | 25951172 | TGATGGATA | TGATGGATA |
| CM-Os159 | chr12 | 2792067 | 2800658 | CCAAGGAC | CCAAGGAC |
| CM-Os160 | chr12 | 407776 | 410074 | ATCTTGTT | ATCTTGTT |
| CM-Os161 | chr12 | 4109709 | 4114978 | TCTGCATGC | TCTGCATGC |
| CM-Os162 | chr12 | 4306165 | 4325390 | TTTCTTGTT | TTTCTAGTT |
| CM-Os163 | chr12 | 5808632 | 5816952 | GGTAGTTAT | GGTAGTTAT |
| CM-Os164 | chr12 | 715459 | 718418 | TGCGACCATT | TGCGACCATT |
| CM-Os165 | chr12 | 769299 | 774812 | CCCGGGTGGA | CCCGGGTGGA |
| CM-Os166 | chr12 | 8174380 | 8179559 | CTAAAAGA | CTAAAAGA |
| CM-Os167 | chr12 | 9444694 | 9452650 | TCTCGGCG | TCTCGGCG |
