# Supplementary material for: Nested Insertions and Accumulation of Indels Are Negatively Correlated with Abundance of Mutator-Like Transposable Elements in Maize and Rice
Source: PLoS One. 2014 Jan 27;9(1):e87069. doi: 10.1371/journal.pone.0087069 (PMC3903597; doi:10.1371/journal.pone.0087069)
Supplement: Table S3 — Pairs of candidate coding-MULEs from maize (CM-Zm) and rice (CM-Os) used in calculating indels. (DOC) [file pone.0087069.s003.doc]

Table S3. Pairs of candidate coding-MULEs from maize (CM-Zm) and rice (CM-Os) used in calculating indels.

| 1^st^ coding-MULE | 2^nd^ coding-MULE |
| --- | --- |
| CM-Zm094 | CM-Zm437 |
| CM-Zm133 | CM-Zm339 |
| CM-Zm135 | CM-Zm421 |
| CM-Zm158 | CM-Zm312 |
| CM-Zm206 | CM-Zm373 |
| CM-Zm223 | CM-Zm345 |
| CM-Zm274 | CM-Zm310 |
| CM-Zm453 | CM-Zm516 |
| CM-Zm235 | CM-Zm360 |
| CM-Zm351 | CM-Zm380 |
| CM-Zm137 | CM-Zm435 |
| CM-Zm094 | CM-Zm182 |
| CM-Zm080 | CM-Zm199 |
| CM-Zm481 | CM-Zm496 |
| CM-Zm275 | CM-Zm222 |
| CM-Zm033 | CM-Zm424 |
| CM-Zm196 | CM-Zm453 |
| CM-Zm312 | CM-Zm473 |
| CM-Zm185 | CM-Zm519 |
| CM-Zm376 | CM-Zm443 |
| CM-Zm178 | CM-Zm292 |
| CM-Zm031 | CM-Zm062 |
| CM-Zm396 | CM-Zm487 |
| CM-Zm275 | CM-Zm389 |
| CM-Zm055 | CM-Zm502 |
| CM-Zm192 | CM-Zm396 |
| CM-Zm049 | CM-Zm294 |
| CM-Zm254 | CM-Zm296 |
| CM-Zm158 | CM-Zm473 |
| CM-Zm003 | CM-Zm466 |
| CM-Zm145 | CM-Zm355 |
| CM-Zm436 | CM-Zm527 |
| CM-Zm048 | CM-Zm502 |
| CM-Zm420 | CM-Zm466 |
| CM-Zm004 | CM-Zm376 |
| CM-Zm021 | CM-Zm026 |
| CM-Zm004 | CM-Zm443 |
| CM-Zm145 | CM-Zm221 |
| CM-Zm248 | CM-Zm520 |
| CM-Zm078 | CM-Zm021 |
| CM-Os141 | CM-Os142 |
| CM-Os223 | CM-Os467 |
| CM-Os389 | CM-Os390 |
| CM-Os040 | CM-Os437 |
| CM-Os201 | CM-Os437 |
| CM-Os437 | CM-Os474 |
| CM-Os266 | CM-Os269 |
| CM-Os326 | CM-Os334 |
| CM-Os350 | CM-Os370 |
| CM-Os249 | CM-Os266 |
| CM-Os008 | CM-Os010 |
| CM-Os266 | CM-Os313 |
| CM-Os229 | CM-Os458 |
| CM-Os076 | CM-Os433 |
| CM-Os230 | CM-Os322 |
| CM-Os394 | CM-Os437 |
| CM-Os110 | CM-Os272 |
| CM-Os431 | CM-Os437 |
| CM-Os053 | CM-Os289 |
| CM-Os289 | CM-Os380 |
| CM-Os110 | CM-Os437 |
| CM-Os128 | CM-Os134 |
| CM-Os238 | CM-Os298 |
| CM-Os081 | CM-Os157 |
| CM-Os136 | CM-Os381 |
| CM-Os078 | CM-Os435 |
| CM-Os098 | CM-Os224 |
| CM-Os262 | CM-Os351 |
| CM-Os238 | CM-Os354 |
| CM-Os176 | CM-Os245 |
| CM-Os063 | CM-Os255 |
| CM-Os090 | CM-Os216 |
| CM-Os139 | CM-Os202 |
| CM-Os238 | CM-Os284 |
| CM-Os136 | CM-Os452 |
| CM-Os238 | CM-Os260 |
| CM-Os194 | CM-Os405 |
| CM-Os217 | CM-Os429 |
| CM-Os276 | CM-Os452 |
| CM-Os134 | CM-Os327 |
| CM-Os021 | CM-Os144 |
| CM-Os223 | CM-Os300 |
| CM-Os103 | CM-Os125 |
| CM-Os271 | CM-Os283 |
| CM-Os224 | CM-Os371 |
| CM-Os176 | CM-Os448 |
| CM-Os103 | CM-Os209 |
| CM-Os283 | CM-Os345 |
| CM-Os162 | CM-Os310 |
| CM-Os163 | CM-Os165 |
| CM-Os109 | CM-Os236 |
| CM-Os351 | CM-Os382 |
| CM-Os262 | CM-Os382 |
| CM-Os010 | CM-Os189 |
| CM-Os136 | CM-Os308 |
| CM-Os109 | CM-Os237 |
| CM-Os237 | CM-Os432 |
| CM-Os237 | CM-Os306 |
| CM-Os220 | CM-Os305 |
| CM-Os400 | CM-Os452 |
| CM-Os136 | CM-Os443 |
| CM-Os131 | CM-Os283 |
| CM-Os136 | CM-Os472 |
| CM-Os283 | CM-Os448 |
| CM-Os276 | CM-Os283 |
| CM-Os236 | CM-Os375 |
| CM-Os018 | CM-Os060 |
| CM-Os060 | CM-Os109 |
| CM-Os103 | CM-Os193 |
| CM-Os103 | CM-Os112 |
| CM-Os283 | CM-Os460 |
| CM-Os136 | CM-Os233 |
| CM-Os102 | CM-Os136 |
| CM-Os022 | CM-Os331 |
| CM-Os218 | CM-Os283 |
| CM-Os135 | CM-Os421 |
| CM-Os103 | CM-Os132 |
| CM-Os236 | CM-Os400 |
| CM-Os108 | CM-Os236 |
| CM-Os127 | CM-Os136 |
| CM-Os004 | CM-Os018 |
| CM-Os010 | CM-Os340 |
